# Quantifying Prescribed‐Fire Smoke Exposure Using Low‐Cost Sensors and Satellites: Springtime Burning in Eastern Kansas

**DOI:** 10.1029/2023GH000982

**Published:** 2024-03-28

**Authors:** Olivia Sablan, Bonne Ford, Emily Gargulinski, Melanie S. Hammer, Giovanna Henery, Shobha Kondragunta, Randall V. Martin, Zoey Rosen, Kellin Slater, Aaron van Donkelaar, Hai Zhang, Amber J. Soja, Sheryl Magzamen, Jeffrey R. Pierce, Emily V. Fischer

**Affiliations:** ^1^ Department of Atmospheric Science Colorado State University Fort Collins CO USA; ^2^ National Institute of Aerospace Hampton VA USA; ^3^ NASA Langley Research Center Hampton VA USA; ^4^ Department of Environmental and Chemical Engineering Washington University in St. Louis St. Louis MO USA; ^5^ Department of Journalism and Media Communication Colorado State University Fort Collins CO USA; ^6^ National Oceanic and Atmospheric Administration College Park MD USA; ^7^ Department of Environmental and Radiological Health Sciences Colorado State University Fort Collins CO USA; ^8^ I.M. Systems Group at NOAA College Park MD USA

**Keywords:** air quality, smoke, PM2.5

## Abstract

Prescribed fires (fires intentionally set for mitigation purposes) produce pollutants, which have negative effects on human and animal health. One of the pollutants produced from fires is fine particulate matter (PM_2.5_). The Flint Hills (FH) region of Kansas experiences extensive prescribed burning each spring (March‐May). Smoke from prescribed fires is often understudied due to a lack of monitoring in the rural regions where prescribed burning occurs, as well as the short duration and small size of the fires. Our goal was to attribute PM_2.5_ concentrations to the prescribed burning in the FH. To determine PM_2.5_ increases from local burning, we used low‐cost PM_2.5_ sensors (PurpleAir) and satellite observations. The FH were also affected by smoke transported from fires in other regions during 2022. We separated the transported smoke from smoke from fires in eastern Kansas. Based on data from the PurpleAir sensors, we found the 24‐hr median PM_2.5_ to increase by 3.0–5.3 μg m^−3^ (based on different estimates) on days impacted by smoke from fires in the eastern Kansas region compared to days unimpacted by smoke. The FH region was the most impacted by smoke PM_2.5_ compared to other regions of Kansas, as observed in satellite products and in situ measurements. Additionally, our study found that hourly PM_2.5_ estimates from a satellite‐derived product aligned with our ground‐based measurements. Satellite‐derived products are useful in rural areas like the FH, where monitors are scarce, providing important PM_2.5_ estimates.

## Key Points

- The Flint Hills of Kansas are impacted by prescribed burning each spring; there were 2.1 million acres burned in 2022
- Particulate matter (PM_2.5_) concentrations substantially increased on days impacted by smoke from fires in eastern Kansas compared to days without smoke
- PM_2.5_ from smoke reaches a maximum overnight in eastern Kansas; this time period is not captured by satellite observations

## 1 Introduction

Exposure to fine particulate matter (PM_2.5_) from anthropogenic sources (e.g., traffic or industrial sources) is associated with a suite of negative health effects including increased risk of cardiopulmonary emergency department visits, hospitalizations, medication use, and mortality (Cohen et al., 2017; Dockery, 2001; Pope, 2000). In the United States, wildfires are a large source of PM_2.5_ in the summertime and fall (Dennison et al., 2014; Ford et al., 2018; Johnston et al., 2012; O’Dell et al., 2019, 2021). There are a number of recent studies that have demonstrated numerous health effects due to wildfire smoke (Abdo et al., 2019; Alman et al., 2016; Gan et al., 2017). However, substantially less research has focused on the health effects of exposure to PM_2.5_ from prescribed fires.

Prescribed burning has distinct regionally specific timing; within the U.S. prescribed burning generally occurs in the spring and fall, when the risk of prescribed fires becoming wildfires is lower than in the summer (Brey et al., 2018; Kaulfus et al., 2017; O’Dell et al., 2021). Depending on the ecosystem, prescribed burns are used to decrease wildfire risk (Francos & Úbeda, 2021; Kolden, 2019; Rosen et al., 2023), manage landscapes (Francos & Úbeda, 2021), and maintain biodiversity (Knapp et al., 2009; Nippert et al., 2021). Because prescribed fires are often smaller, of short duration, and occur where there is a lack of in situ air quality monitoring, the smoke from prescribed burning is often not measured.

Springtime burning occurs annually in the Flint Hills (FH) region of Kansas (Kansas Department of Health and Environment, 2022; Mohler & Goodin, 2012; Rosen et al., 2023; Scholtz et al., 2020). The FH region extends from northeastern Kansas to northeastern Oklahoma. The counties that are considered to be in the FH as defined by the Kansas Department of Health and Environment (KDHE) include 21 counties (Kansas Department of Health and Environment, 2022). These counties are sparsely populated (Figure S1 in Supporting Information S1; US Census Bureau, 2023) with an average county population of about 28,000 per county.

Prescribed fires in the FH decrease wildfire risk, increase nutrition of vegetation for livestock (Duncan et al., 2021), control invasive grass species (Alexander et al., 2021; Ditomaso et al., 2006), and maintain ecosystem diversity, resilience, and health in the tall grass prairie (Bruckerhoff et al., 2020; Ohlenbusch & Hartnett, 2000). The tall grass prairie is a rare ecosystem and is threatened by woody encroachment. Prescribed burning is practiced for abatement of this encroachment (Abrams, 1988; Morford et al., 2022; Short et al., 2019). Prescribed fires also have cultural significance in the FH (Rosen et al., 2023); for example, indigenous people practiced prescribed burning in the Great Plains to hunt for bison (Roos et al., 2018), and residents of many identities participate in social activities surrounding the burn season.

The health effects of exposure to smoke from prescribed fires are less studied than those from wildfires (Pennington et al., 2023) used fire radiative power from satellites to estimate smoke exposure in Kansas, and they found an increase in asthma emergency department visits in Kansas from exposure to smoke from prescribed burning. However, this study had a high potential for misclassification of exposure. The exposure estimates relied on modeling and satellite observations due to the lack of in situ measurements in Kansas. The correlation with their PM_2.5_ estimates, and the ground‐based sensors was 0.33. Because the prescribed fires in the FH are often small and short‐lived, more monitoring is essential for quantification of the PM_2.5_ smoke contribution to better understand associations with health outcomes.

The areas that practice prescribed burning are often away from urban centers, and, consequently, further away from regulatory monitors (Kelp et al., 2022; US EPA, 2015), so the local PM_2.5_ impacts from the prescribed burns are not fully quantifiable using only these monitors. Hence, smoke impacts in this region are still uncertain. This study seeks to understand the impact of prescribed burning in the FH on PM_2.5_ concentrations. Our goal is to attribute PM_2.5_ concentrations to smoke from fires in the FH.

## 2 Methods

We deployed in situ low‐cost PurpleAir monitors in eastern Kansas between March and May 2022 to supplement the surface US Environmental Protection Agency Air Quality System (EPA AQS) PM_2.5_ measurements during the spring burning season. We combined these data with satellite observations to identify the location of fires, distinguish the presence of smoke in the atmospheric column, and confirm elevated aerosol loading. Satellite products used in this work include MODIS burned area, Geostationary Operational Environmental Satellite Aerosol Optical Depth (GOES AOD) (Zhang et al., 2020), and National Oceanic and Atmospheric Administration Hazard Mapping System (HMS) (NOAA HMS) smoke plumes and fire hotspots. We discuss each of these data sets and their use in the following sections.

### 2.1 In Situ Monitoring

**Figure 1:**
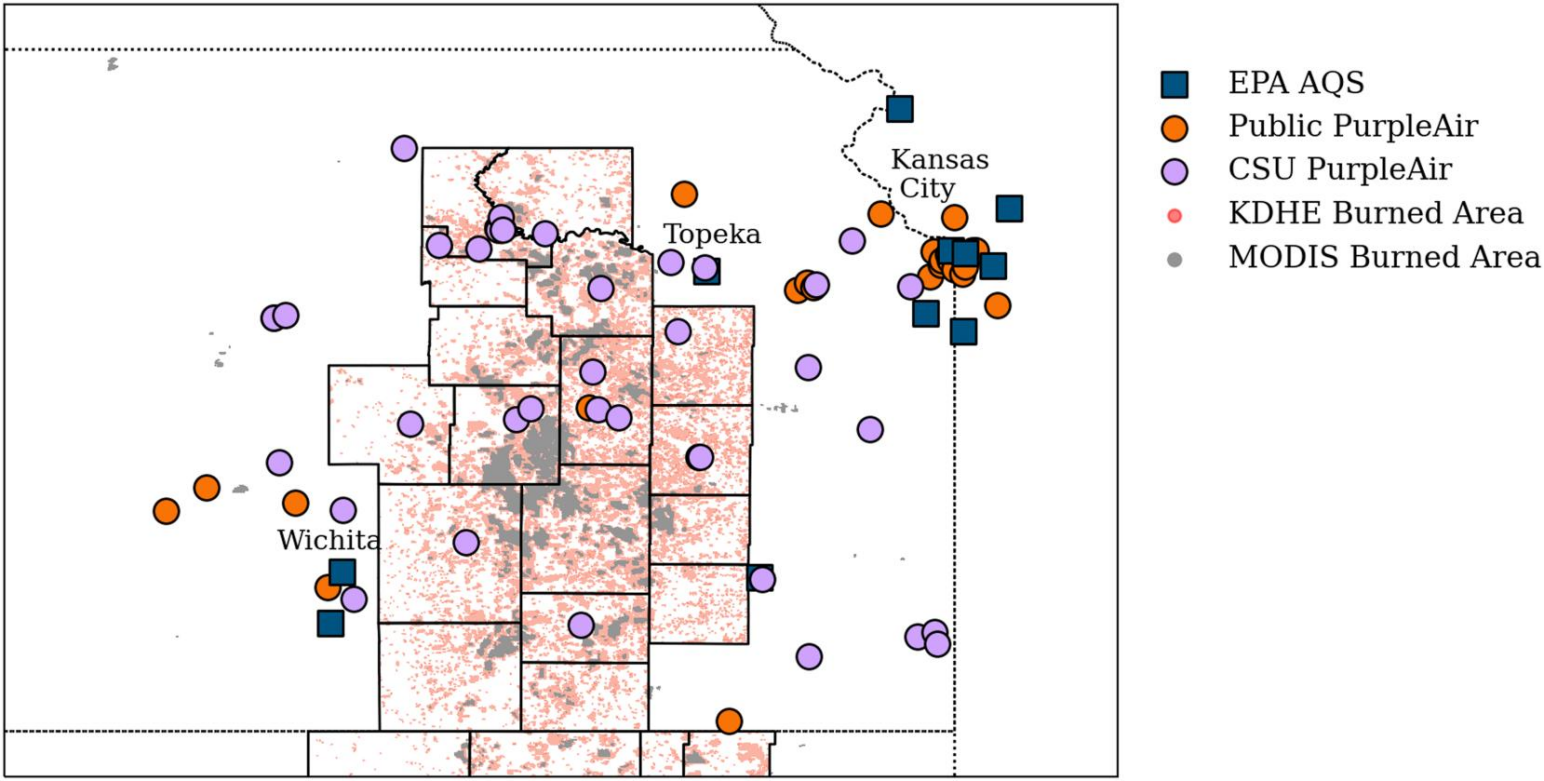
Locations of in situ PM_2.5_ monitors and burned area from Kansas Department of Health and Environment and Moderate Resolution Imaging Spectroradiometer in eastern Kansas. County lines are included for the Flint Hills counties.

We used PM_2.5_ measurements from the EPA AQS and the PurpleAir real‐time air quality‐monitoring networks to study eastern Kansas smoke impact. At the time of this study, there were 11 regulatory monitors in the area of study (Figure 1). These monitors were primarily located in more populated urban centers (Topeka, KS; Wichita, KS; and Kansas City, MO) outside of the counties where most of the prescribed burns occur. We used hourly and daily PM_2.5_ measurements from the EPA AQS. We used measurements from both regulatory methods: Federal Reference Method and Federal Equivalent Method (FEM) monitors (Parameter Code 88101). FRMs and FEMs monitors use recognized and standardized approaches to measure PM_2.5_ concentrations in compliance with regulatory standards. We removed the monitoring site in Picher, Oklahoma (40‐115‐9007) through the entirety of our analysis due to lack of data during the campaign timeframe.

To supplement the ground‐based PM_2.5_ measurements from the EPA AQS regulatory monitors, we used PM_2.5_ concentrations from PurpleAir. PurpleAir are low‐cost sensors (∼$300 USD per unit) that estimate PM_2.5_ concentrations every 2 min. PurpleAir sensors contain two Plantower (PMS‐5003) light‐scattering particle sensors (channel A and B), measuring at 680 ± 10 nm. For this study, we used the raw (“CF1”) PM_2.5_ estimates (aerodynamic diameter of <2.5 μm). The sensors also include a BOSCH BME280 to measure pressure, temperature, and humidity. PurpleAir sensors have been evaluated in laboratory and field studies (Barkjohn et al., 2021; Jaffe et al., 2022; Malings et al., 2020; Tryner et al., 2020) and were found to have relatively high precision but relatively lower accuracy, especially when there were high temperatures and humidity. To improve the accuracy of the PM_2.5_ concentrations from PurpleAir, we applied a correction factor (full description in Section 2.1.1).

#### 2.1.1 PurpleAir Monitor Deployment

The existing 28 PurpleAir sensors that have publicly available data in eastern Kansas and western Missouri are primarily located away from the burning (Figure 1). Thus, we recruited participants to deploy 38 PurpleAir monitors throughout eastern Kansas. Most of the prescribed burning of tallgrass prairies is conducted in April. The Kansas Administrative Regulation places restrictions on burning activities, such as of waste and debris, in the 16 regulated counties of the FH during the month of April (Kansas Administrative Regulation, 2012); thus, there may be an increase in these other types of burns during March. We had participants deploy sensors for March‐May 2022 to capture the entire burning season. These sensors provided spatial coverage throughout the FH where the majority of prescribed burning occurs in Kansas (Figure 1). We deployed 20 sensors in FH counties and 18 sensors in surrounding counties of eastern Kansas. Volunteer participants hosted the PurpleAir sensors at residences, campuses, and public buildings.

#### 2.1.2 PurpleAir Data Quality Procedures

We conducted pre‐campaign quality control of the PurpleAir sensors from October ‐ December 2021 in Fort Collins, CO. The Plantower lasers are factory calibrated by PurpleAir. Prior to deployment in KS, we confirmed that each sensor produced accurate PM_2.5_ estimates across both channels and in comparison to a FEM monitor (GRIMM EDM 180, Ainring, Germany). Sensors were co‐located (<5 m) with the GRIMM monitor for a period of 8–12 days. We conducted the quality control analysis on 10‐min averages of the raw PurpleAir data (“CF1”). PM_2.5_ concentrations during this analysis ranged from 0 to 49.2 μg m^−3^. When comparing channel A and channel B within each PurpleAir device, the correlation between channels averaged 0.99; the lowest *R* ^2^ was 0.84 (Figure S2 in Supporting Information S1). The sensor with the lowest correlation between channels (sensor 1361028) was deemed suitable for deployment because the average PM_2.5_ from the two instrument channels was consistent with the GRIMM monitor observations. During the calibration tests, the mean absolute difference between channels ranged across the devices from 0.18 to 1.2 μg m^−3^. The 10‐min channel average of each sensor was strongly correlated to the 10‐min average of all sensors (mean *R* ^2^ = 0.99; mean bias = 0.05%). The mean *R* ^2^ between the 10‐min average PurpleAir and the GRIMM PM_2.5_ concentrations was 0.68 (Figure S3 in Supporting Information S1). The low *R* ^2^ was likely due to the lack of variability in concentrations during the co‐location time period as the average mean absolute error only ranged from 1.9 to 2.6 μg m^−3^. These findings are consistent with past studies (Magi et al., 2020; Sayahi et al., 2019), and will improve with the application of a correction factor, detailed below. We acknowledge our pre‐campaign testing may not be representative of the field conditions; however, our testing informed us of any issues with individual PurpleAir sensors prior to field deployment.

Before analysis of the campaign data, we performed a quality check on the raw PM_2.5_ concentrations from PurpleAir. We took 10‐min averages of all measurements. We then removed data with the following conditions: (a) temperature >65°C (0.005% of observations), (b) relative humidity >100% (0.0001% of observations), (c) channel disagreement >10% from the average of the two channels or 10 μg m^−3^ in the absolute difference between the channels (3.3% of observations), and (d) measurements >500 μg m^−3^ (0.0017% of observations).

We applied the Barkjohn et al. (2021) correction factor to the quality checked PurpleAir field measurements. This correction factor reduces bias in PurpleAir PM_2.5_ by scaling based on the measured concentrations and relative humidity. The US Environmental Protection Agency developed the correction factor using measurements from 53 PurpleAir sensors co‐located with regulatory monitors across 16 states, including one site in Kansas. This national correction factor was suitable for our analysis. Because we removed data with concentrations >500 μg m^−3^, we did not use the correction factor for extreme smoke concentrations (>570 μg m^−3^) (Barkjohn et al., 2021). Due to continuously erroneous humidity observations for one sensor (PurpleAir 1361083), we used humidity observations from a nearby PurpleAir sensor to calculate the PM_2.5_ correction factor. After we applied the correction factor, some observations with high humidity and low concentrations became negative. All negative concentrations after the correction factor were set to 0 μg m^−3^ (1.87% of observations). We found good agreement between one of the deployed PurpleAir monitors and a nearby (∼1.2 km) regulatory monitor in the field, with a correlation of 0.75 (Figure S4 in Supporting Information S1).

We calculated daytime and nighttime averages of the PM_2.5_ concentrations for the hours of 7:00 to 19:00 and 19:00 to 7:00 Central Daylight Time (CDT), respectively. The time associated with observations after 13 March 2022 were shifted an hour later to account for daylight savings.

### 2.2 Satellite Products

To investigate the extent of burning we obtained MODIS burned area and the Kansas Department of Health and the Environment Moderate Resolution Imaging Spectroradiometer (KDHE‐MODIS) burned area product (Mohler & Goodin, 2012; Scholtz et al., 2020). Satellite products were essential for the identification of transported smoke. For our smoke designation (see full description in Section 2.3), we used GOES AOD and the NOAA Hazard Mapping System (HMS) fire and smoke plume product (National Oceanic and Atmospheric Administration, 2022a, 2022b; Rolph et al., 2009; Ruminski et al., 2023). We also compared our in situ PM_2.5_ results to two satellite‐derived PM_2.5_ products (van Donkelaar et al., 2021; Zhang & Kondragunta, 2021).

#### 2.2.1 Fire and Smoke Detection

We used smoke plumes and fire hotspots from NOAA HMS (National Oceanic and Atmospheric Administration, 2022a, 2022b; Rolph et al., 2009; Ruminski et al., 2023). HMS uses polar and geostationary satellite observations along with other sources to digitize smoke plumes and fire locations. We included all smoke plumes and fire hotspots in the HMS data set for each day of the study period. We assessed whether the smoke plumes overlapping monitors were from a small local fire or from long‐range transport using the horizontal extent of the HMS plume. Large plumes that cover greater regions (i.e., all of eastern Kansas—330 km × 250 km) are not typically from local fires.

We used the MODIS/Terra + Aqua Direct Broadcast Burned Area Monthly L3 Global 500 m SIN Grid V061 product (Giglio et al., 2021) and the KDHE‐MODIS product for the years 2001–2022 to investigate the timing and extent of the burning in Kansas (Figure 1). Satellite‐based detection systems have difficulty detecting small short‐lasting fires (e.g., Hu et al., 2016); thus, the KDHE develops seasonal burned area estimates for the FH counties using the MODIS MCD64A1 burned area product that is then also verified by field observations (Mohler & Goodin, 2012; Scholtz et al., 2020). Since burning in the FH is voluntarily reported, and therefore incomplete, KDHE instead made field observations to improve upon the MODIS MCD64A1 burned area product. The KDHE‐MODIS product does not provide fine temporal resolution, but rather gives a seasonal average of burned area. KDHE found that MODIS underpredicted the burned area by 28% in the FH (Figure 1).

#### 2.2.2 AOD and Satellite‐Derived PM_2.5_

To help investigate whether or not the smoke was from fires outside KS, we used GOES‐16 Advanced Baseline Imager (ABI) bias‐corrected AOD data at 550 nm (Zhang et al., 2020). The AOD measurements have a 5‐min temporal resolution and a 2 km spatial resolution. When the smoke designation (i.e., local vs. transported smoke) was more challenging to determine, we used daily averages of AOD to help our decision‐making process (Section 2.3).

AOD is a measure of the aerosol loading in the atmospheric column and not a direct measurement of surface air quality. Thus, to compare satellite observations to our surface observations, we used two satellite based PM_2.5_ products. The NOAA GOES Geographically Weighted Regression (GWR) PM_2.5_ product (Zhang & Kondragunta, 2021) provides hourly and daily estimates of surface PM_2.5_ derived from the Suomi National Polar‐Orbiting Partnership Visible Infrared Imaging Radiometer Suite and GOES‐16 ABI along with ground‐based PM_2.5_ concentrations from AirNow (https://www.airnow.gov/). The algorithm derives a regression relationship between PM_2.5_ and AOD using GWR from the PM_2.5_ and AOD. The surface PM_2.5_ is then estimated from AOD using the derived relationship. To compare hourly PM_2.5_ concentrations from in situ measurements and hourly PM_2.5_ from the NOAA GOES GWR product, we applied the smoke designations (Section 2.3) by using the PM_2.5_ from the satellite product only at the locations of the monitors. The categorization for each day was applied to all hours during that day.

We also compared our ground‐based PM_2.5_ measurements to the van Donkelaar et al. (2021) Monthly Hybrid PM_2.5_ product (V5GL04). For 2022 data, this product combines the daily best estimates of AOD retrievals from several products including the Multi‐angle Imaging SpectroRadiometer (MISR), MODIS Dark Target, MODIS Deep Blue, and MODIS Multi‐Angle Implementation of Atmospheric Correction. The combined best‐estimate AOD is related to ground‐level PM_2.5_ concentrations using output from the GEOS‐Chem chemical transport model and subsequently statistically fused with in situ measurements from EPA AQS (i.e., blue squares in Figure 1) through GWR to provide 24‐hr monthly mean surface PM_2.5_. We compared seasonal averages (March‐May) of our ground‐based measurements to the derived PM_2.5_.

### 2.3 Smoke Assessment

Each daily measurement was categorized by the type of smoke impacting the monitoring site (Table 1) using a decision tree (Figure S5 in Supporting Information S1). The decision process considered the following variables: HMS smoke plumes and fire hotspots, in situ PM_2.5_ measurements from PurpleAir and EPA AQS sites, in situ coarse mode concentrations (PM_10_‐PM_2.5_) from EPA AQS sites, daily wind speed, categorization of the previous day, and proximity to an urban setting. A detailed description of the decision tree is available in Supporting Information S1.

**Table 1:** Description of the Daily Category Assigned to Each Monitoring Location, the Number of Days in Each Category (Monitor Days), and the 10th and 90th Percentile Number of Days Per Monitor

Daily designation	Definition	Total number of monitor days	Average number of days per monitor	10th percentile number of days per monitor	90th percentile number of days per monitor
*Smoke‐free*	No smoke was identified in the atmospheric column	1,918	26	12	33
*Eastern Kansas Smoke Impacted* (*EK*_*only*_)	Smoke originating from fires only in the eastern Kansas region was in the atmospheric column	271	4	1	7
*Transported Smoke Impacted*	Smoke originating from fires only outside of eastern Kansas was in the atmospheric column	2,613	35	23	43
*EK + T Smoke Impacted*	Smoke originating from fires both outside and within eastern Kansas was in the atmospheric column	1,080	15	8	23

For each day at each measurement site, we separated the conditions into four categories: *Smoke‐free*, *EK*_*only*_ *Smoke Impacted, Transported Smoke Impacted*, and *EK + T Smoke Impacted* (Table 1). We designated the day to be *Smoke‐free* when we determined no smoke to be in the atmospheric column over a given monitor. We designated a day as *Eastern Kansas Smoke Impacted (EK*_*only*_ *)* when there was smoke in the atmospheric column originating from fires in the eastern Kansas region, but there was no smoke from fires outside of this region. We categorized days as *Transported Smoke Impacted* when there was smoke in the atmospheric column originating from fires outside of eastern Kansas. When we identified days where smoke originated from fires both outside and within eastern Kansas, we classified these days as *Eastern Kansas and Transported Smoke Impacted (EK + T).* In our analysis, we combined EK_only_ and EK + T as *EK*_*all*_ to capture all of the days where monitors were impacted by smoke from fires within eastern Kansas, regardless of if there was also transported smoke. For our categorization, we only considered monitors within eastern Kansas and Kansas City, Missouri. This included 75 total PurpleAir sensors and EPA AQS monitors. We considered daily PM_2.5_ concentrations from 7 to 7 a.m. CDT to include the impact of evening burning and smoke traveling downwind that often elevated PM_2.5_ concentrations in the region into the following morning.

**Figure 2:**
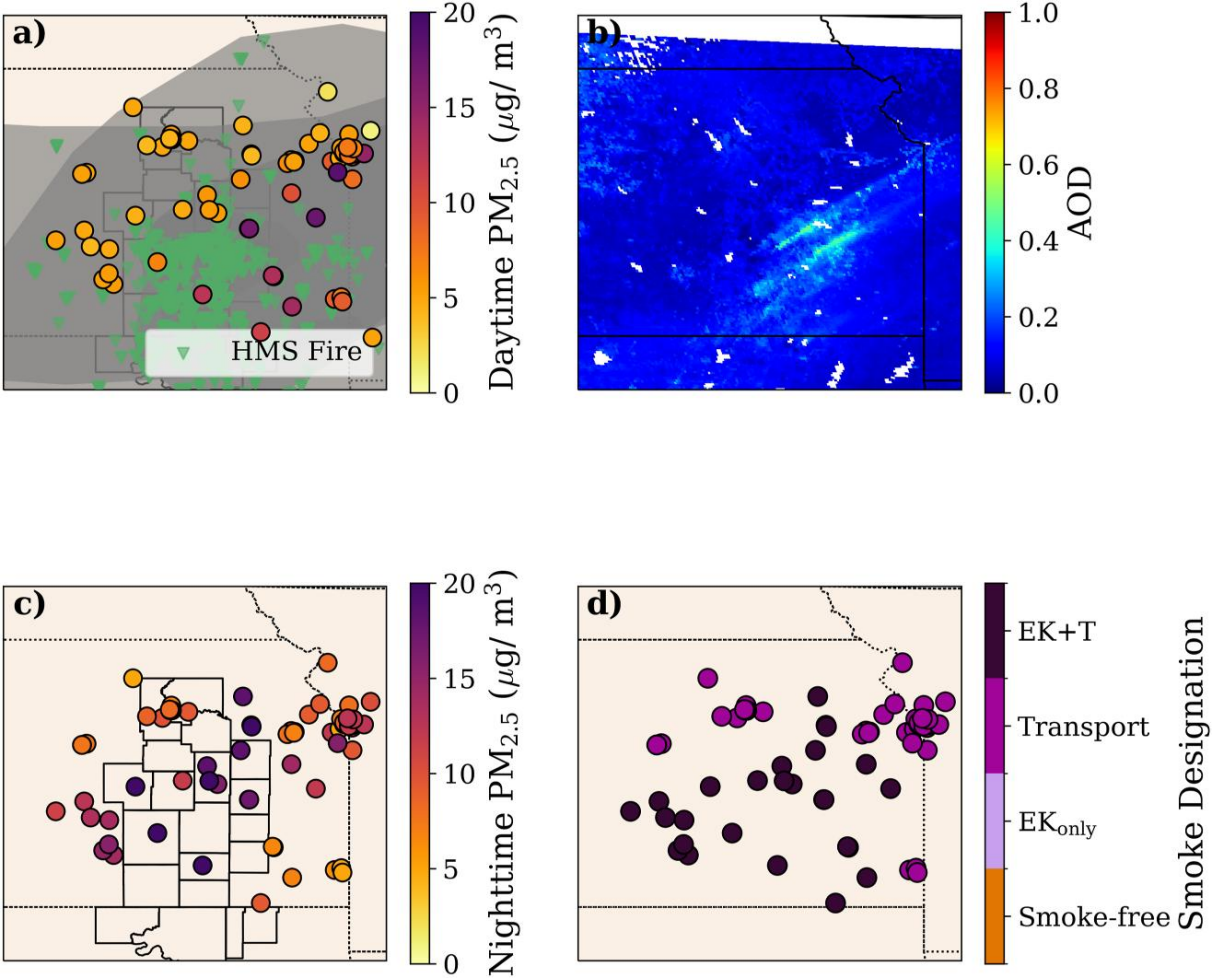
To determine the daily smoke designation, we used (a) daytime (7 a.m.–7 p.m. Central Daylight Time (CDT)) PM_2.5_ concentrations, (b) mean GOES AOD (550 nm) (c) nighttime (7 p.m.–7 a.m. CDT) PM_2.5_ concentrations. The resulting smoke designations for 14 April 2022 are shown in (d).

Figure 2 shows an example of our smoke designation for 14 April 2022 alongside some of the parameters that we used to perform the categorization (HMS smoke plumes and fire hotspots, daytime PM_2.5_ concentrations, daily AOD, and nighttime PM_2.5_). For this case, HMS smoke plumes overlapped all of the monitors of interest and PM_2.5_ concentrations were elevated (>10 μg m^−3^) during the daytime in southeastern Kansas. HMS fire hotspots were present to the west of these monitors; thus, we categorized the monitors for this day as either *Transported Smoke Impacted* or *EK + T Impacted*.

## 3 Results

### 3.1 Overview of the 2022 Burning Season

We investigated the timing and extent of the 2022 FH burning season using the KDHE burned area product and the MODIS burned area product. The KDHE product estimates the burned area within the 21 FH counties. We therefore only included the MODIS product burned area estimates for the FH counties in our analysis. We also examined the number of days that smoke was observed in the atmospheric column using smoke plumes from HMS.

#### 3.1.1 The Extent and Timing of Burning in 2022

**Figure 3:**
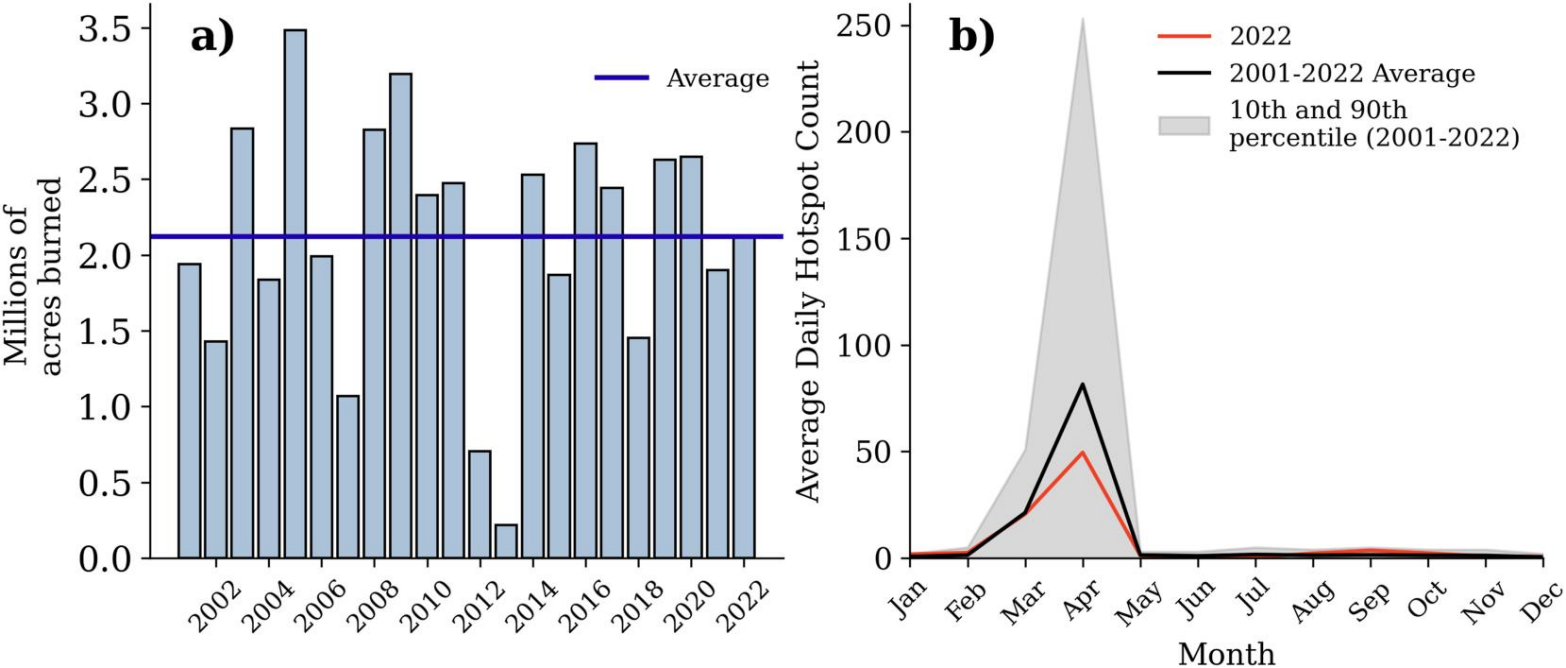
(a) Acres burned from the Kansas Department of Health and Environment burned area product and (b) average daily hotspot count from Moderate Resolution Imaging Spectroradiometer in the Flint Hills counties from 2001 to 2022.

The KDHE‐MODIS burned area product reported 2,112,759 acres total acres burned in the FH counties in 2022 (Figure 3). There is large interannual variability of burned area; 217,377 acres burned in 2013 compared to almost 3.5 million acres in 2005. The 2022 burned area was very similar to the average (2,123,419 acres) across the time period 2001–2022, suggesting that it was not an anomalous year in terms of burning.

To investigate the timing of the burning, we used the hotspot fire detection from the MODIS product to assess when the burning occurred in the FH in 2022. We counted hotspots only within the designated FH counties to be consistent with the KDHE‐MODIS product. The maximum average daily hotspot counts in 2022 occurred in April (50 daily hotspots), consistent with past years (2001–2022) (Figure 3). As mentioned previously, there are restrictions placed on types of burning other than tallgrass prairies in April. The smaller increase in fires in March may be due to an increase in waste or debris burning before restrictions begin in April.

#### 3.1.2 Transported Smoke

**Figure 4:**
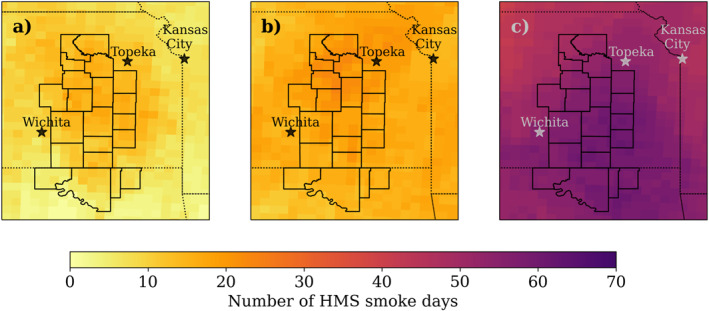
Count of days with an Hazard Mapping System plume in the atmospheric column on a 15 × 15 km degree grid for spring (March‐May) (a) 2020, (b) 2021, and (c) 2022.

The FH can be impacted by smoke from local burning and from transported smoke. Because wildfire season in the west typically occurs in late spring and summer; in most years, springtime smoke is primarily due to local burning. However, eastern Kansas was significantly impacted by transported smoke during spring 2022. Figure 4 compares the number of days there were NOAA HMS smoke plumes over our study region (HMS smoke days) in 2020, 2021, and 2022. Smoke was frequently present in the atmospheric column over eastern Kansas and northern Oklahoma during the 2022 burning season (91 total days) with a minimum of 40 and a maximum of 62 days (calculated for total map area shown in Figure 4). More smoke was present in the atmospheric column during 2022 than prior years, with a spatial average of 53 HMS smoke days in 2022 over eastern Kansas and northern Oklahoma, compared to 16 in 2021 and only 9 in 2020.

The increased number of days with smoke over eastern Kansas in 2022 was due to transported smoke from fires located outside of the region shown in Figure 4. Smoke was transported from fires located in Oklahoma, Nebraska, Texas, and New Mexico. However, New Mexico experienced two of the largest wildfires in the state's history spring 2022; the Hermits Peak and Calf Canyon fires persisted for months and burned 1,382 km^2^ (Tunby et al., 2023). The Hermits Peak fire started on 6 April 2022 and the Calf Canyon fire started on 19 April 2022. These fires merged and were 100% contained by 21 August 2022 (Tunby et al., 2023). This wildfire overlapped with two of the 3 months of our campaign. We observed smoke during this event transported to the FH area (Figures S8 and S9 in Supporting Information S1). Separating the transported smoke impact from *EK* smoke impact was important in isolating the impact of local FH fires on PM_2.5_ concentrations.

### 3.2 Attributing Smoke in Eastern Kansas to Local Versus Distant Fires

**Figure 5:**
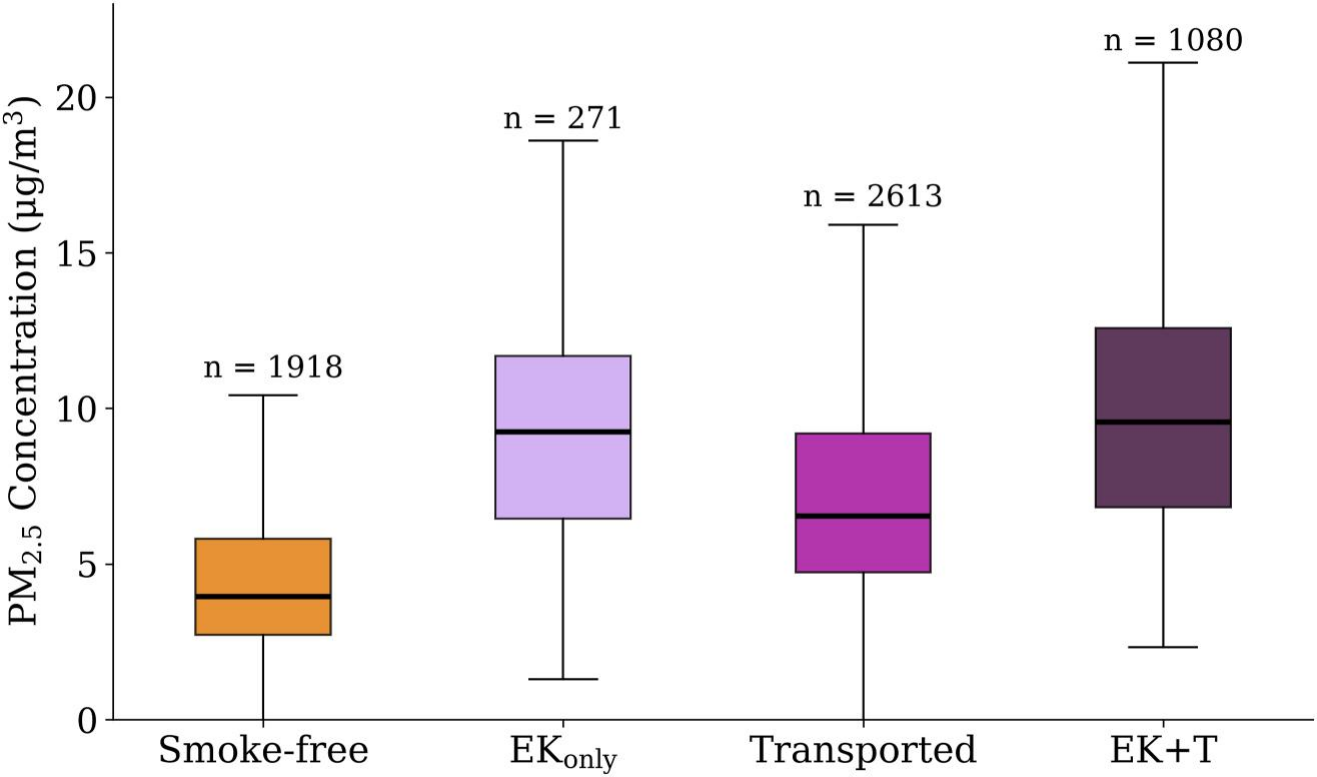
Daily averages (7–7 a.m. Central Daylight Time) of PM_2.5_ concentrations for *Smoke‐free*, *EK*_*only*_, *Transported*, and *EK + T* days during the spring burning season of 2022. Outliers have been excluded and the bold line represents the median for each category. The edges of the box represent the first and third quartiles. The whiskers are ±1.5 times the interquartile range from the first and third quartile. Number of monitor days in each category is shown in Table 1.

With the goal of attributing PM_2.5_ surface concentrations to smoke, we followed the method outlined in Section 2.3 and categorized the daily smoke impact for each monitor. We then separated daily average (7–7 a.m. CDT) PM_2.5_ concentration by the smoke designation. In Figure 5, we show the distribution of daily PM_2.5_ concentrations for all monitors and all days. PM_2.5_ concentrations were higher on EK_only_ days compared to *Smoke‐free* days (median of 9.2 vs. 4.0 μg m^−3^); however, there were few days categorized as *EK*_*only*_ (an average of four *EK*_*only*_ *Smoke Impacted* days for each monitor (Table 1)). The smoke designations show a median PM_2.5_ concentration increase of 5.3 μg m^−3^ on days impacted by smoke from fires in eastern Kansas compared to days without smoke.

There was an increase in PM_2.5_ during *Transported Smoke* days, relative to *Smoke‐free* days, with a median concentration of 6.6 μg m^−3^ on *Transported Smoke* days. The tail of the distribution suggests that on some days, transported smoke had little impact on surface concentrations, implying that smoke was aloft. There was a similar, though slightly higher, distribution of PM_2.5_ concentrations on *EK + T Smoke Impacted* days compared to the distribution of *EK*_*only*_ *Smoke Impacted* days. Because these distributions are similar and because there were few *EK*_*only*_ days at each monitor location; for the rest of the analysis, we combined *EK*_*only*_ and *EK + T* days into one designation (*EK*_*all*_).

We assessed the statistical significance of the smoke category distributions of PM_2.5_ concentrations using a Mann‐Whitney *U* test which is a non‐parametric test used for independent samples (Mann & Whitney, 1947). We conducted this test to evaluate if our methods separated smoke impacts into distinct designations. Our null hypothesis is that the distributions of PM_2.5_ for each smoke designations are identical. The alternative hypothesis is that PM_2.5_ concentrations are distributed differently for our smoke designations. When comparing the distributions for *EK*_*only*_ and *EK + T*, we observed the highest *p*‐value (*p*‐value = 0.04), whereas all other distributions yielded smaller *p*‐values (*p*‐values < 0.001). Although all *p*‐values were representative of statistically different distributions with 95% confidence (*p*‐values < 0.05), the substantially larger *p*‐value for the comparison of *EK*_*only*_ and *EK + T* further supports our choice to combine *EK*_*only*_ and *EK + T* into *EK*_*all*_. By combining these categories, we acknowledge there may be some impact from transported smoke included in our attribution of smoke PM_2.5_ to local EK fires. In the following analysis, we provide other estimates to mitigate this limitation.

**Figure 6:**
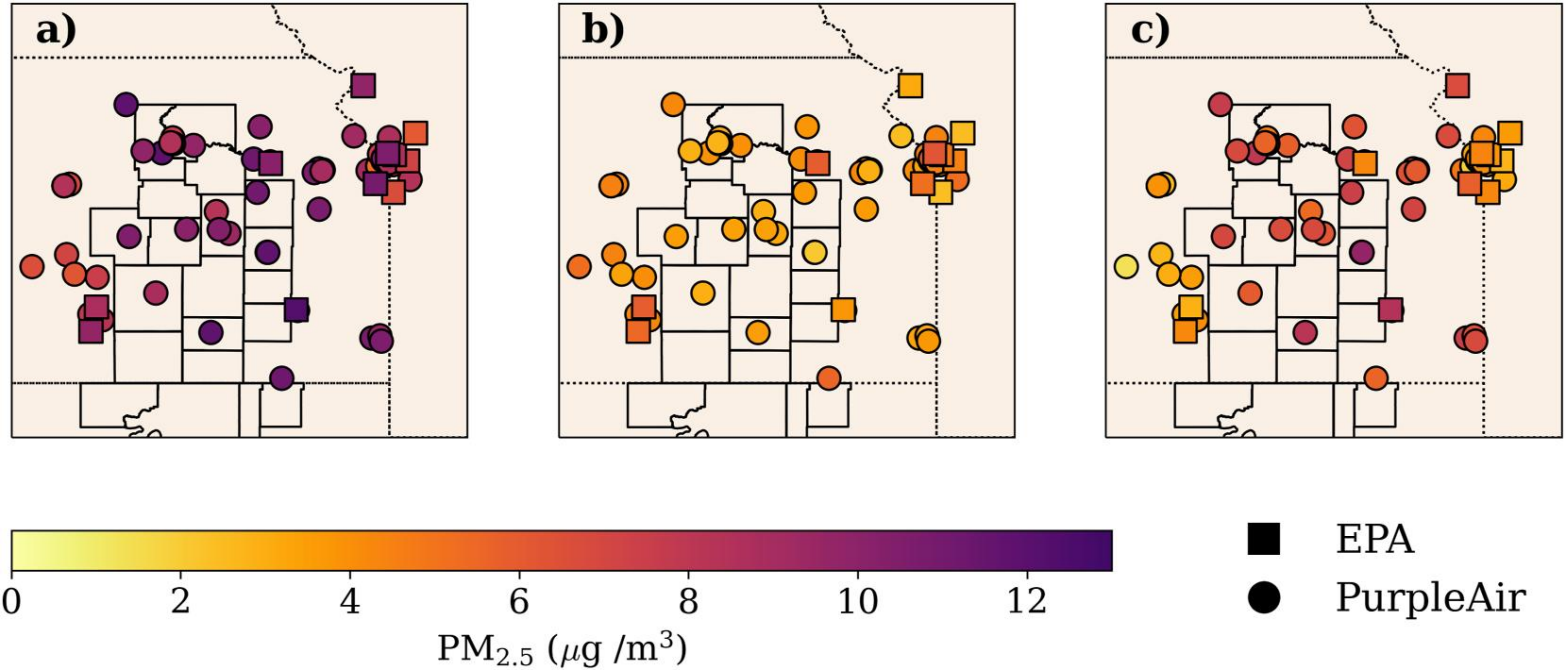
(a) Median daily (7–7 a.m. Central Daylight Time) PM_2.5_ concentration on EK_all_ days, (b) *Smoke‐free* days, and (c) the difference between the medians at each site between March‐May. Marker shape indicates monitor type. Monitors with less than 10 days in either *EK*_*all*_ or *Smoke‐free* impacted days were not included in order to remove spatial bias of monitors with few observations in these smoke designations.

During the 2022 burning season, we observed substantial spatial variability in the impact of smoke on PM_2.5_ across eastern Kansas. In Figure 6, we show the median concentration at each monitor on *EK*_*all*_ days, on *Smoke‐free* days, and the difference. PM_2.5_ concentrations during *EK*_*all*_ *Smoke Impacted* days ranged from 5.1 to 12.3 μg m^−3^ across all monitors for the 2022 prescribed burning season. PM_2.5_ concentrations during *Smoke‐free* days were relatively low across the region, ranging from 2.0 to 6.2 μg m^−3^ with the maximum occurring in the Kansas City region. We considered the contribution of smoke from eastern Kansas fires to PM_2.5_ concentrations to be the difference between PM_2.5_ concentrations on *EK*_*all*_ *Smoke Impacted* days and smoke‐free days.

The largest contribution of smoke to PM_2.5_ concentrations occurred in the FH counties and directly east of the FH. Throughout eastern Kansas, the median contribution was 5.0 μg m^−3^; and within the FH counties, the median contribution was 6.7 μg m^−3^. For this analysis, we combined the days with local smoke impact (*EK*_*only*_) and days with local and transported smoke impact (*EK + T*). With this approach, we may have included some impact from transported smoke into our attribution of smoke PM_2.5_ to local EK fires. If we subtract the median PM_2.5_ during *Transported Smoke Impacted* days from *EK + T* days, the median PM_2.5_ concentration attributable to local smoke would be 3.0 μg m^−3^. We can compare to the median smoke contribution to PM_2.5_ concentrations on days with only local smoke impact (*EK*_*only*_‐*Smoke‐free*) of 5.3 μg m^−3^. However, the number of *EK*_*only*_ days was low (average of 4 days per monitor), and the distributions of PM_2.5_ concentrations on EK + T and *EK*_*only*_ days were similar. Transported smoke likely had a small impact on *EK + T* days.

The contribution of local smoke to the springtime (March, April, May) was calculated by multiplying the median local‐smoke contribution (*EK*_*all*_ days) to PM_2.5_ concentration by the fraction of springtime days affected by local smoke for each monitor. We found the contribution of local smoke to the springtime (March, April, May) median to be 1.9 μg m^−3^. *EK*_*all*_ days accounted for 26% of the total study period. Under the assumption that rangeland burning is entirely during this season (Figure 3), and we assume no burning during other months, then the median contribution of local smoke to the annual average is 0.5 μg m^−3^. If we consider monitors only within the FH counties, the springtime contribution is 2.6 μg m^−3^, and the annual contribution is 0.6 μg m^−3^. With a higher contribution in the FH, residents in these counties are more affected by local burning compared to residents in other counties in Kansas. We also compared our estimate to the smoke PM_2.5_ product from O’Dell et al. (2021), where surface PM_2.5_ attributable to smoke (from all types of fires: prescribed, agricultural, and wildfire) is estimated using surface monitors and NOAA HMS for the years of 2006–2018. O’Dell et al. (2021) found the mean annual contribution of smoke to PM_2.5_ in the FH to be between 0.24 and 0.41 μg m^−3^. We estimated the annual contribution to be up to about twice that of this smoke product, showing the smoke from prescribed fires may be underestimated in the O’Dell et al. smoke product.

#### 3.2.1 Comparing the Diurnal Cycle of PM_2.5_ Across Regions

During the 2022 burning season, we observed substantial temporal variability in the impact of smoke on PM_2.5_ across eastern Kansas. We found the largest contribution of smoke to PM_2.5_ concentrations to occur in the FH during the evening. In our investigation of the temporal impact of smoke, we examined PM_2.5_ concentrations from ground‐based measurements throughout the day, separated by smoke designation. *Smoke‐free* days did not have a significant diurnal cycle; however, during *EK*_*all*_ *Smoke Impacted* days, there was often a diurnal cycle, with PM_2.5_ concentrations increasing in the afternoon and building throughout the evening and overnight. This is consistent with (a) emissions continuing into the evening as FH fires smolder and (b) the reduction of the planetary boundary layer height before sunset, such that the smoke is trapped at the surface when wind speeds are low until the next day. For these reasons, we often considered daily averages of the ground‐based concentrations to be from 7 to 7 a.m. CDT (as used in the aforementioned analysis) to estimate the contribution of smoke to PM_2.5_ in the evening.

**Figure 7:**
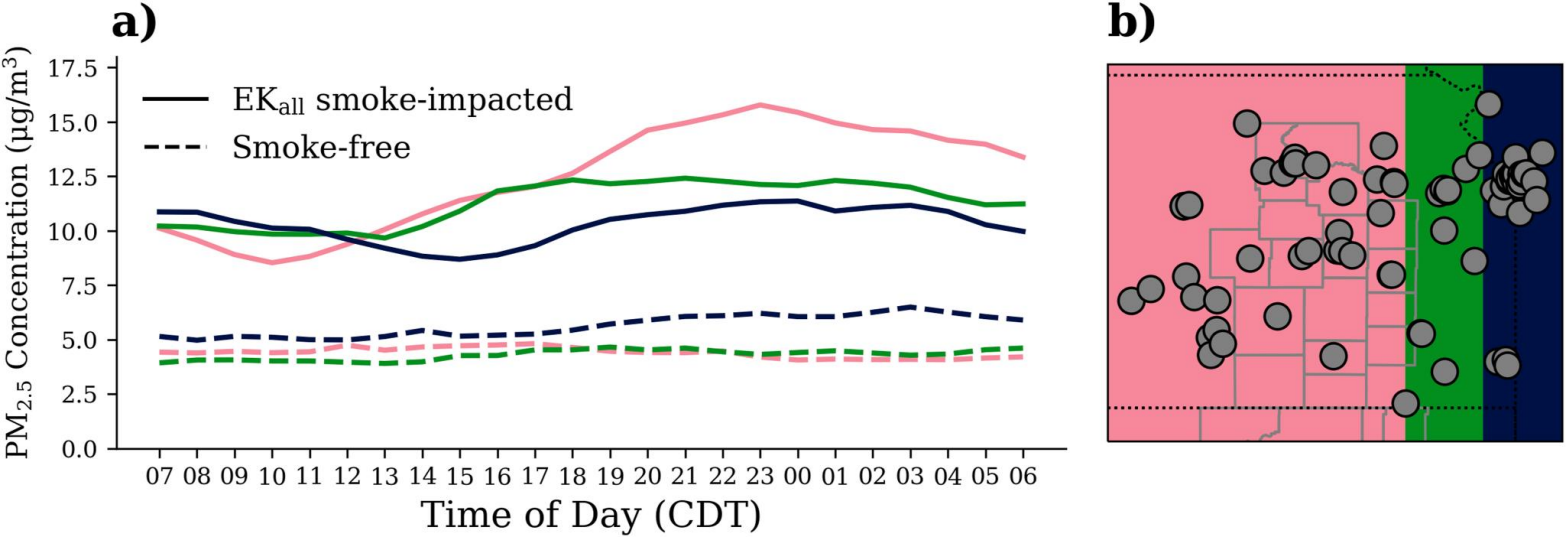
(a) Hourly average of PM_2.5_ across all ground‐based monitors for March‐May 2022 for *Smoke‐free* days and *EK*_*all*_ *Smoke Impacted* days for three different longitudinal slices shown in (b). Smoke designation was applied to every hour of the respective day.

We separated monitors longitudinally into the following groups to evaluate the diurnal cycle downwind of the fires (Figure 7): monitors west of the FH and in the FH (pink), monitors in the area directly downwind of the FH (green), and monitors further downwind in Pittsburg, KS and the Kansas City metropolitan area (dark blue). Much less burning occurs in the counties east of the FH. The monitors west of the FH had about half of *EK*_*all*_ *Smoke Impacted* observations as the monitors in the FH counties. Most of the *EK*_*all*_ observations for the monitors west of the FH occurred during the Cottonwood Complex wildfire in Hutchinson, KS in March. For these reasons, we grouped the monitors west of the FH and in the FH.

PM_2.5_ across all sites was not significantly higher in the evening during *Smoke‐free* days. For the monitors west of the FH and in the FH, there was a mean increase from 8.5 to 15.8 μg m^−3^ during *EK*_*all*_ *Smoke Impacted* days when comparing 10:00 to 23:00 CDT. There was a smaller increase on *EK*_*all*_ *Smoke Impacted* days in the easternmost region (dark blue), with about 1.2 μg m^−3^ of a difference. We observe a diurnal cycle, with EK_all_ PM_2.5_ concentrations increasing in the evening for the monitors most impacted by the FH burning (pink). The monitors directly to the east of the FH and in Kansas City had a muted evening peak, in comparison to the FH monitors. Although there was not as significant of a diurnal cycle here, concentrations were higher on smoke impacted days versus smoke‐free days.

### 3.3 In Situ and Satellite‐Derived PM_2.5_ Comparison

**Figure 8:**
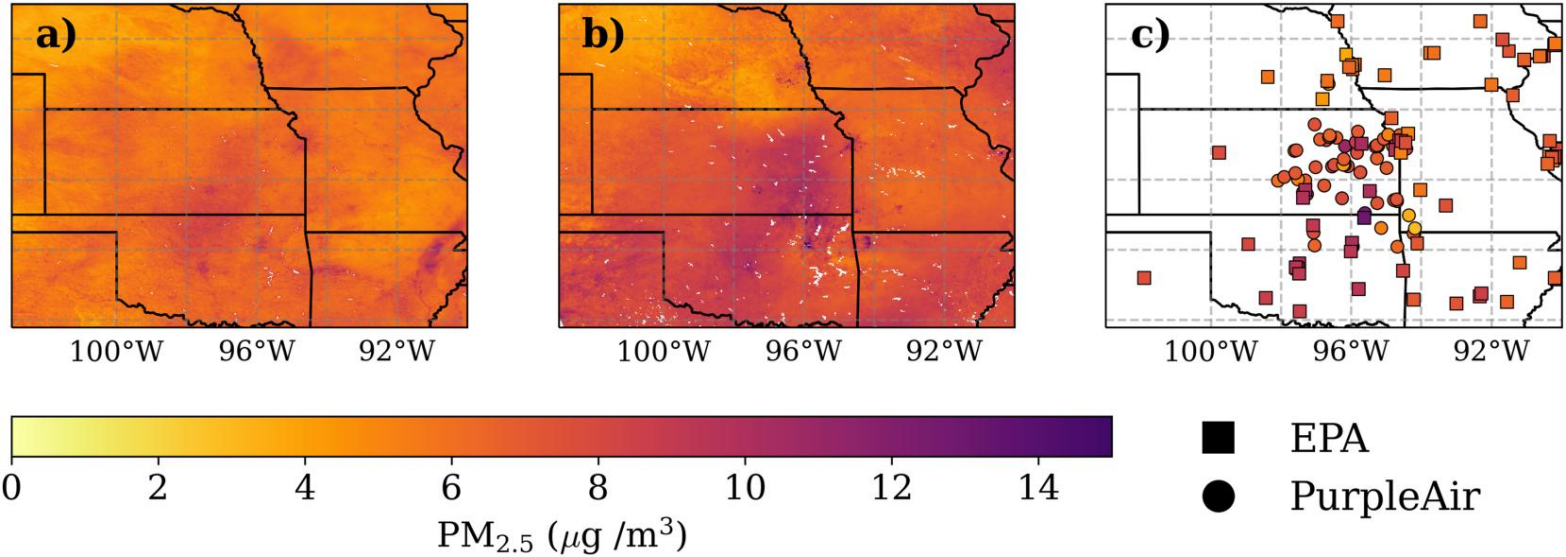
Comparison of (a) average 2022 PM_2.5_ concentrations in March‐May from the V5GL04 Monthly Hybrid product, (b) the satellite derived product from NOAA GOES Geographically Weighted Regression, and (c) in situ measurements from PurpleAir in the Flint Hills and EPA AQS for the entire mapped region. Marker shape indicates monitor type.

To assess the accuracy of surface PM_2.5_ estimates in the FH from satellite‐derived products, we compared two of these products (van Donkelaar et al., 2021; Zhang & Kondragunta, 2021) with the surface PM_2.5_ measurements. The V5GL04 Monthly Hybrid PM_2.5_ product has monthly estimates; thus, we first compare seasonal averages rather than sub‐daily estimates. Both the satellite products (NOAA GOES GWR and V5GL04 Monthly Hybrid) and in situ measurements (Figure 8 and Figure S9 in Supporting Information S1) confirm elevated PM_2.5_ concentrations in the FH region compared to the surrounding area during the 2022 springtime burning season. When comparing ground‐based monitors in eastern Kansas to each product, the mean absolute error against the surface monitors was 1.9 μg m^−3^ (NOAA GOES GWR) and 1.2 μg m^−3^ (V5GL04 Monthly Hybrid). There were higher PM_2.5_ concentrations estimated in the FH area compared to the surrounding area in the NOAA GOES GWR product (hourly estimates) than the V5GL04 Monthly Hybrid product. The mean normalized bias between the seasonal average of the satellite products and in situ measurements in eastern Kansas were 29% (NOAA GOES GWR) and 2% (V5GL04 Monthly Hybrid) (Figure S10 in Supporting Information S1). Kansas City exhibited more disagreement between satellite products and surface measurements, with a bigger range of mean percent difference in both products. This may be due to the diverse spatial variability due to urban sources. The mean absolute error increased to 2.2 μg m^−3^ (NOAA GOES GWR) and 1.3 μg m^−3^ (V5GL04 Monthly Hybrid) in Kansas City. Despite fair overall mean agreement, the local correlation was low between the satellite products and the surface measurements, with *R* ^2^ = 0.02 (V5GL04 Monthly Hybrid) and *R* ^2^ = 0.03 (NOAA GOES GWR) (Figure S10 in Supporting Information S1). This shows that surface monitors are critical for capturing fine‐scale gradients and variability. Satellite products give important insight to large‐scale surface PM2.5 patterns, but in situ measurements are critical for capturing PM2.5 at fine spatial scales, as with smoke from small, prescribed fires in the FH region.

We compared past years of satellite‐derived PM_2.5_ seasonal averages and found some interannual variability in the estimated PM_2.5_ from the satellite products (Figure S11 in Supporting Information S1). Both satellite products estimated PM_2.5_ in the FH to be lower in 2020 compared to 2021 and 2022, although the acreage burned was above average in 2020 and below average in 2021 (Figure 3). As with 2022, the estimated PM_2.5_ in the FH during the burning season for 2020 and 2021 was higher in the NOAA GOES GWR product than the V5GL04 Monthly Hybrid PM_2.5_ product for the 2020–2022 burning seasons.

**Figure 9:**
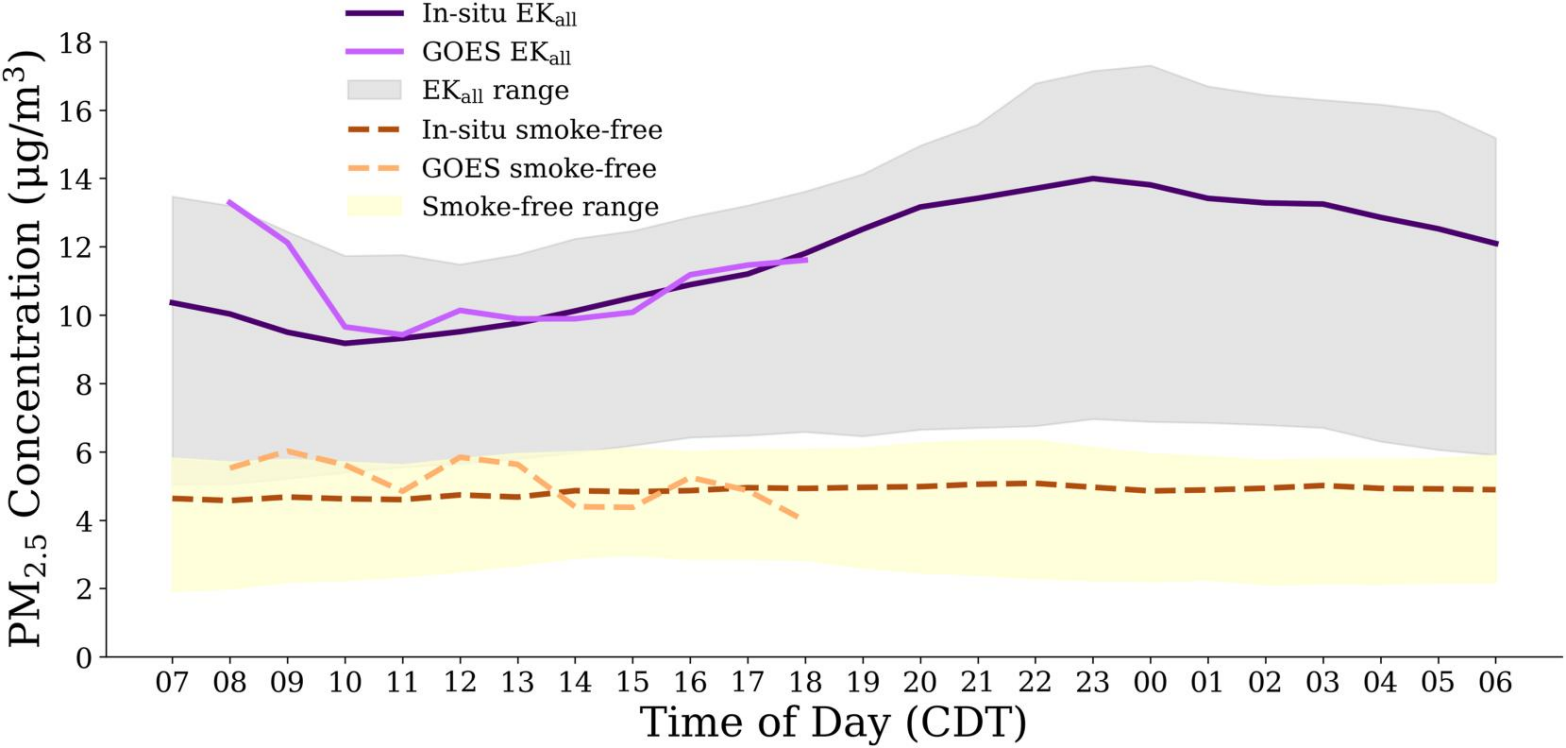
Hourly average of PM_2.5_ across all ground‐based monitors for March‐May 2022 separated by *Smoke‐free* days and *EK*_*all*_ *Smoke Impacted* days compared to the PM_2.5_ derived concentrations from NOAA GOES Geographically Weighted Regression. The 25th and 75th percentiles are shaded for *Smoke‐free* and *EK*_*all*_ days. The derived PM2.5 concentrations at 7:00 and 19:00 have been removed due a limited number of observations. There were only 70 observations at 7:00 and 142 at 19:00 compared to an average number of observations for all other time periods of 299. The two removed hours were likely impacted by low solar zenith angles, resulting in a smaller number of accurate observations and lower accuracy when observations exist.

The PM_2.5_ concentration on *EK*_*all*_ *Smoke Impacted* days was consistently higher versus *Smoke‐free* days for both the PM_2.5_ product from the NOAA GOES GWR product (Zhang & Kondragunta, 2021) and the ground‐based measurements (Figure 9). The difference between the average PM_2.5_ concentrations in all in situ monitors for *EK*_*all*_ days and smoke‐free days during the satellite observation period (8:00–18:00) was 5.4 μg m^−3^, and the difference of the average for PM_2.5_ concentrations from the satellite product was 5.7 μg m^−3^. The satellite product does not measure the overnight peak in PM_2.5_ that is observed by the in situ monitors. However, the satellite and in situ estimates have a mean percent difference of 6.5% across the entire day where there are overlapping observations for *EK*_*all*_ days and 8.2% for *Smoke‐free* days. The NOAA GOES GWR product hourly PM_2.5_ estimates were consistent with our *EK*_*all*_ and *Smoke‐free* in situ hourly averages.

## 4 Conclusions and Limitations

In this study, we deployed 38 PurpleAir monitors in the FH region of Kansas to assess the impact of smoke from prescribed burning during the spring burning season (March through May) 2022. The burning season was impacted by smoke transported from outside of the region in addition to the smoke from the local prescribed fires. To investigate the impact of local fires alone, we separated transported smoke from eastern Kansas smoke using a decision tree. We compared our in situ measurements to two satellite PM_2.5_ products (NOAA GOES GWR and the V5GL04 Monthly Hybrid).

Median PM_2.5_ concentration on *Smoke‐free* days during spring 2022 was 4.0 μg m^−3^, in comparison to 9.4 μg m^−3^ for any day with smoke originating in eastern Kansas (*EK*_*all*_). PM_2.5_ concentrations were highest in the FH counties and directly to the east in 2022 compared to other areas of eastern Kansas. The diurnal cycle of PM_2.5_ on days impacted by smoke from local fires (increasing in the evening time) was also most pronounced in the FH region.

Hourly PM_2.5_ concentrations during the day from the NOAA GOES GWR product were similar to our ground‐based measurements on days with local fires and on *Smoke‐free* days. During local‐fire days, there was an overnight peak in PM_2.5_ observed with in situ measurements that were unobserved by the satellite product. Thus, satellite products underestimate total PM_2.5_ in the FH, especially at night. We found higher concentrations of PM_2.5_ in the FH area compared to the surrounding areas during the 2022 burning season (March‐May).

Transported smoke introduced uncertainty in our attribution of PM_2.5_ to smoke from local fires. We separated and designated smoke sources using a decision tree. We were motivated by (a) the lack of satellite observations under some conditions (e.g., cloudy days, overnight) and (b) the need to separate smoke from local versus distant fires. However, our decision‐tree process is imperfect, and there are several ways where errors may occur. For example, on days when there were no satellite observations and the ground‐based PM_2.5_ concentrations were low, we likely designated this as a *Smoke‐free* day. If there had been a small smoke impact during this day, we could have underestimated the total average smoke exposure across the season/year (although our estimated smoke concentration on smoke days would be biased high). Also, our decision‐tree used a concentration of 10 μg m^−3^ to separate days where smoke likely impacted monitors versus not, which was especially important in smoke designation when there were no HMS plumes or hotspots. If we chose a higher/lower value than 10 μg m^−3^ for a threshold, we would have fewer/more smoke‐impacted days, leading to a different attribution of smoke to PM_2.5_ concentrations. As there were many other factors in the decision‐tree process, this impact would likely not lead to meaningful differences.

We combined local smoke and transported smoke designations due to the similar distributions in PM_2.5_ concentrations and low number of days with only local smoke. This may have resulted in an overestimation of local smoke impacts. If we subtract the median PM_2.5_ during *Transported Smoke Impacted* days from days with both local and transported smoke (*EK + T*), the median PM_2.5_ concentration attributable to local smoke would be 3.0 μg m^−3^. We compare median smoke contribution to PM_2.5_ concentrations on days with only local smoke impact (*EK*_*only*_‐*Smoke‐free*), and this resulted in a difference of 5.3 μg m^−3^. This indicates that our inclusion of days with local and transported smoke into our calculation of local smoke contribution to PM_2.5_ may have led to an overestimation. However, this would likely be a smaller overestimation (<2 μg m^−3^) as the distributions of our two local smoke designations were statistically similar (*p*‐value > 0.01), so transported smoke likely had a smaller impact on days that also had local smoke. Our alternative estimate of PM_2.5_ attributable to local smoke (3.0 μg m^−3^) provides a lower bound that accounts for this limitation.

PurpleAir sensors have been shown to perform with low bias at low concentrations and high bias at high concentration during our pre‐campaign quality control and in previous work (Sayahi et al., 2019; Tryner et al., 2020). We applied the Barkjohn et al. (2021) correction factor to reduce this bias; however, this is a nation‐wide correction factor and there may be a correction factor more suited for this region and type of fire. Additionally, we acknowledge the bias that we may have introduced when applying the correction factor to our data at the 10‐min average compared to a 24‐hr average. Introducing this correction factor early on may have also resulted in an underestimation of PM_2.5_. The PM_2.5_ concentrations during this campaign were relatively low, with the daily mean concentration of 7.3 μg m^−3^ for March‐May. Based on the comparison between one of our PurpleAir sensors that was located near an EPA AQS site (Figure 4), the underestimation could still be on the order of a few micrograms (mean difference between the corrected PurpleAir sensor and the EPA AQS monitor was −2.4 μg m^−3^).

Prescribed burning practices in the FH of Kansas are unique compared to other prescribed fires across the United States. The main fuel type in the FH is the rare tallgrass prairie, distinguishing it from other regions, like the Northwest (WA, OR, ID) and Southwest US (CA, NV, AZ) where the dominant fuel is evergreen needleleaf forest (Brey et al., 2018). Additionally, the prescribed burning season in the FH typically occurs during the spring months (March‐May), which differs from other regions like Yosemite National Park, CA where the prescribed burning occurs from April to June and September to November (Knapp et al., 2009). Due to the diverse characteristics of prescribed burning practices in the US, we cannot generalize our findings in the FH to other regions. With these differences in mind, it would be valuable to deploy low‐cost monitors to other regions in order to gain a better understanding of the impact of smoke from prescribed fires on PM_2.5_.

Our results indicate that smoke from small, short‐lived, prescribed burns increase ground‐based PM_2.5_ concentrations. Smoke and the associated health effects from prescribed fires should be further investigated in the FH and other areas of the US. Rural communities are often the most affected by prescribed burning, and these regions can lack consistent long‐term federal air quality monitors, which is a finding supported by this work. The health effects of smoke from prescribed burns are also understudied. Satellite‐derived products give estimates of ground‐based concentrations in these areas, but in the FH, there is an increase of smoke from daytime prescribed fires overnight, when satellite observations are limited. To further investigate the impact of smoke in the FH and the effect of health, future work could develop a product of smoke estimates using fused methods. These smoke estimates could be applied to health data to assess the impact.

## Conflict of Interest

The authors declare no conflicts of interest relevant to this study.

## Supporting information

Supporting Information S1

## Data Availability

The smoke designations for each monitor and the monitor locations are available at Sablan et al. (2023). The PurpleAir data from the field deployment can be downloaded from Sablan et al. (2024). The PurpleAir data from public monitors can be accessed through the PurpleAir API, and the details can be found here: https://community.purpleair.com/t/making‐api‐calls‐with‐the‐purpleair‐api/180. The EPA regulatory PM_2.5_ data is publicly available and can be downloaded from https://aqs.epa.gov/aqsweb/airdata/download_files.html. The MODIS MCD64A1 burned area product is available at Giglio et al. (2021). Estimates from the KDHE‐MODIS burned area product are provided at Kansas Department of Health and Environment (2022). The NOAA/NEDIS Hazard Mapping System Smoke Product is available at National Oceanic and Atmospheric Administration (2022b) and the Fire Product is available at National Oceanic and Atmospheric Administration (2022a). The surface PM_2.5_ from the V5GL04 Monthly Hybrid product can be accessed at van Donkelaar et al. (2021). The NOAA GOES GWR PM_2.5_ product is available at Zhang and Kondragunta (2021).
