# Supplementary material for: Quantifying Prescribed‐Fire Smoke Exposure Using Low‐Cost Sensors and Satellites: Springtime Burning in Eastern Kansas
Source: Geohealth. 2024 Mar 28;8(4):e2023GH000982. doi: 10.1029/2023GH000982 (PMC10975953; doi:10.1029/2023GH000982)
Supplement: Supplementary file 1 — Supporting Information S1 [file GH2-8-e2023GH000982-s001.docx]

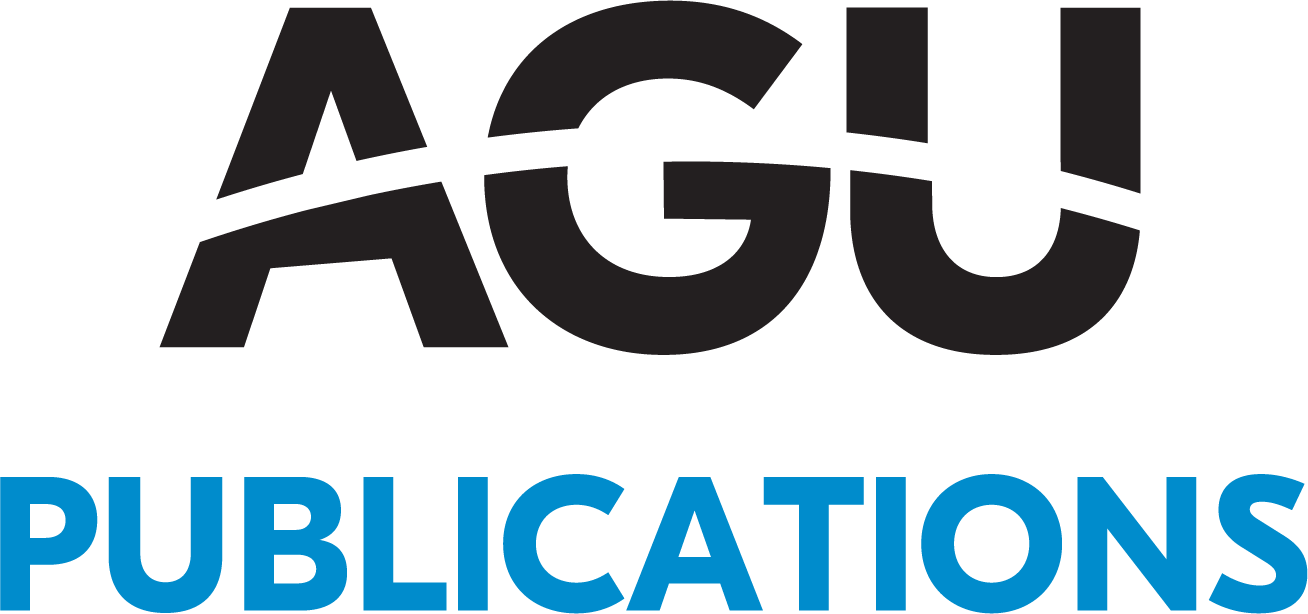


*AGU GeoHealth*

Supporting Information for

**Quantifying prescribed-fire smoke exposure using low-cost sensors and satellites: Springtime burning in Eastern Kansas**

Olivia Sablan^1^, Bonne Ford^1^, Emily Gargulinski^2,3^, Melanie S. Hammer^4^, Giovanna Henery^5^, Shobha Kondragunta^6^, Randall V. Martin^4^, Zoey Rosen^5^, Kellin Slater^7^, Aaron van Donkelaar^4^, Hai Zhang^8^, Amber J. Soja^3^, Sheryl Magzamen^7^, Jeffrey R. Pierce^1^, and Emily V. Fischer^1^

^1^Colorado State University, Department of Atmospheric Science

^2^National Institute of Aerospace

^3^NASA Langley Research Center

^4^Washington University in St. Louis, Department of Environmental and Chemical Engineering

^5^Colorado State University, Department of Journalism and Media Communication

^6^National Oceanic and Atmospheric Administration

^7^Colorado State University, Department of Environmental and Radiological Health Sciences

^8^I.M. Systems Group at NOAA

**Contents of this file**

Text S1 to S11

Figures S1 to S11

**Introduction**

This supplemental material provides additional context and details essential for a comprehensive understanding of various aspects related to our findings. It details the distribution of the Kansas population, pre-deployment quality checks of PurpleAir monitors, the decision-making process for smoke designation, the grouping of monitors for smoke impact assessment, a notable smoke transport event from New Mexico, and a comparison between satellite products and in situ measurements. This comprehensive information delves into the demographic characteristics of the Flint Hills counties, quality control measures for deployed monitors, the intricacies of smoke categorization, and the impact of external smoke events. Additionally, it further compares the two satellite-derived products. Overall, this supplemental material aims to offer a detailed and transparent view of the methodologies and contextual factors shaping the research outcomes.

Text S1.

Kansas Population

The Flint Hills counties in Kansas are Butler, Chase, Chautauqua, Coffey, Cowley, Elk, Geary, Greenwood, Lyon, Marion, Morris, Osage, Pottawatomie, Riley, Wabaunsee, Wilson, and Woodson (Figure 1). These counties are sparsely populated (Figure S1). Woodson County was the most populated in 2022 compared to the other Flint Hills counties, with about 71,000 people. The most populated county in Kansas in 2022 was Wyandotte county, which includes part of the Kansas City metropolitan area. This county included over 600,000 people, more than 8 times the population of Woodson county.


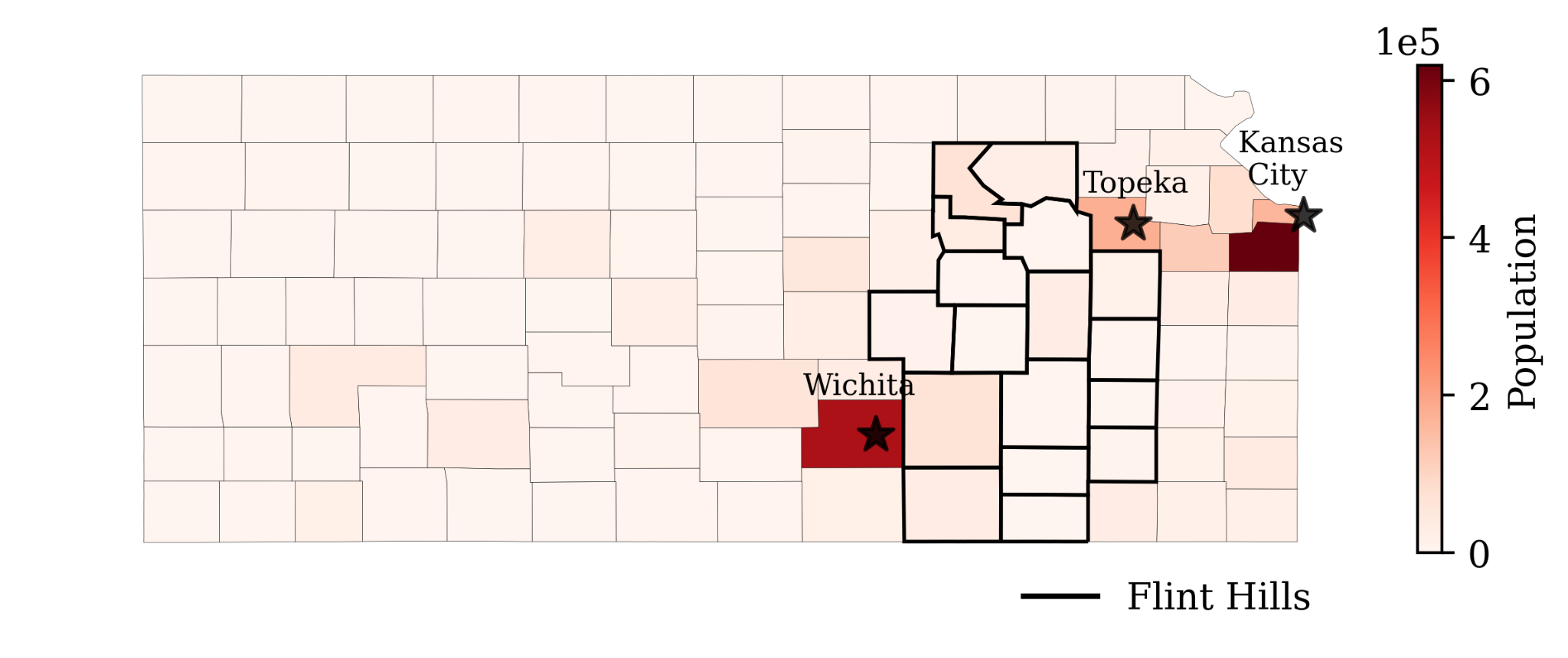


Figure S1. The 2022 population by county, with the Kansas Flint Hills counties outlined (US Census Bureau, 2023a).

Text S2.

PurpleAir pre-deployment quality check

Prior to deploying our PurpleAir monitors to the field, we conducted a quality check of the monitor performance. We installed 44 PurpleAir monitors in Fort Collins, CO. We ran three separate tests to evaluate the sensors. The first group of 19 sensors were evaluated from 28 October 2021 to 04 November 2021. The second group of 19 sensors were evaluated from 11 November 2021 to 18 November 2021. The last group of 8 sensors were evaluated from 20 November 2021 to 01 December 2021. One PurpleAir sensor remained up for the entirety of the testing. We compared the PM_2.5_ concentrations from channel A and channel B of the PurpleAir (Figure S2). The average PM_2.5_ concentration for the first test was 8.14 µg m^-3^. The average PM_2.5_ concentration for the second test was 2.91 µg m^-3^. The average PM_2.5_ concentration for the third test was 7.17 µg m^-3^. The mean correlation between channels for all three testing periods was 0.995.


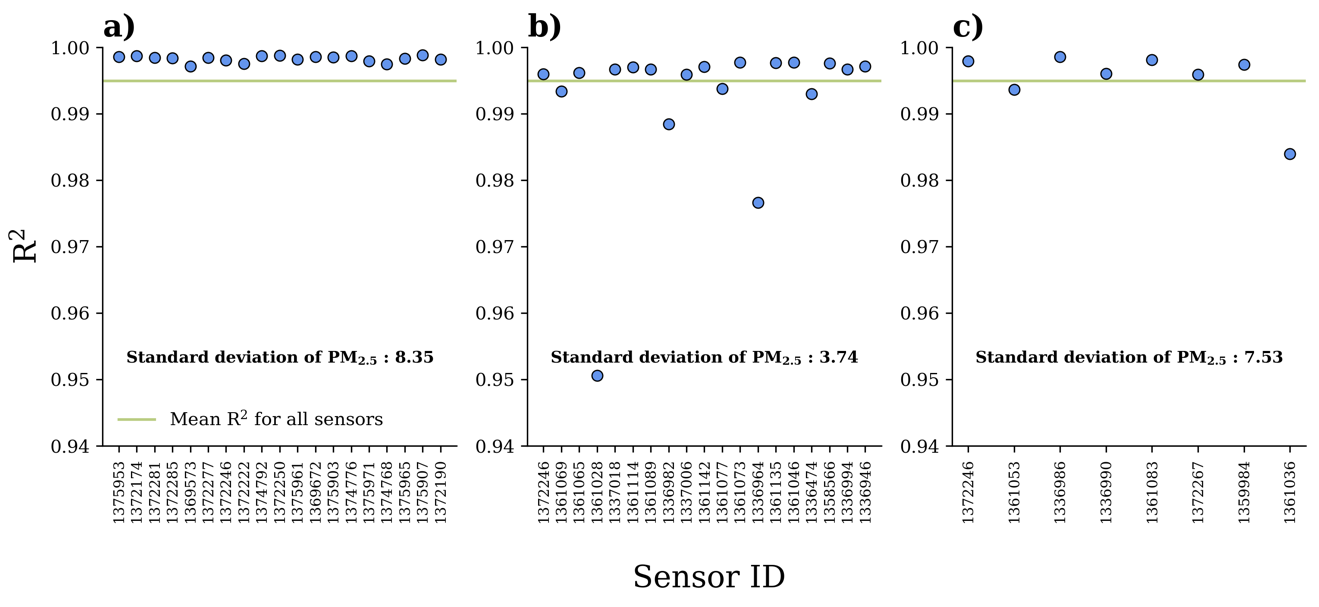


**Figure S2.** Pearson correlation between PurpleAir channel A and channel B in comparison to the average correlation of all sensor channels (horizontal green line) for testing period 1 (a), 2 (b), and 3 (c).

Text S3.

Additionally, we compared the PM_2.5_ concentrations from our PurpleAir sensors to a co-located Federal Equivalent Method (GRIMM) monitor. The GRIMM PM_2.5_ concentrations were compared to the PurpleAir for the three testing groups, over the same time periods we compared the PurpleAir channels (Figure S3). The average correlation for all three tests was 0.68.

**
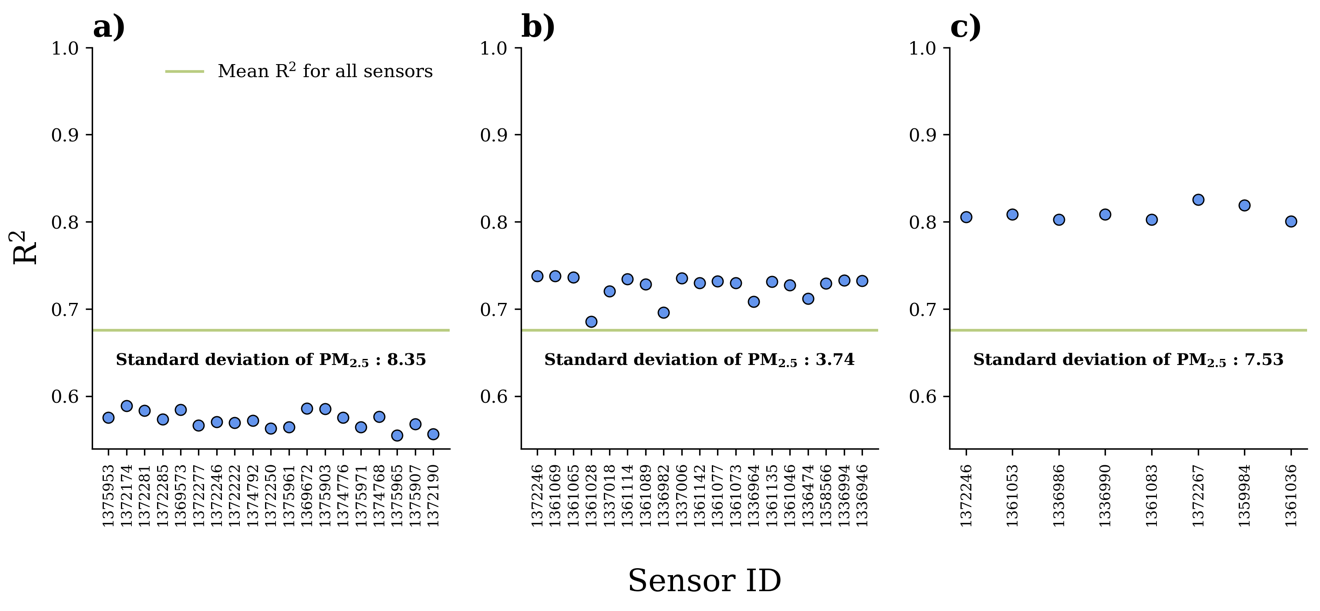
**

**Figure S3.** Pearson correlation between PurpleAir sensors and the GRIMM monitor in comparison to the average correlation of all sensors (horizontal green line) and the GRIMM for testing period 1 (a), 2 (b), and 3 (c).

Text S4.

We compared PM_2.5_ concentrations from a PurpleAir and a regulatory monitor in Chanute, KS (Figure S4). Although we deployed other PurpleAir monitors near regulatory monitors, we compared these monitors because they were about close in proximity (~1.2 km) and because Chanute is not a major metropolitan area. The mean absolute difference between the corrected PurpleAir PM_2.5_ concentrations and the regulatory monitor concentrations was 2.84 µg m^-3^. The correlation was 0.75.


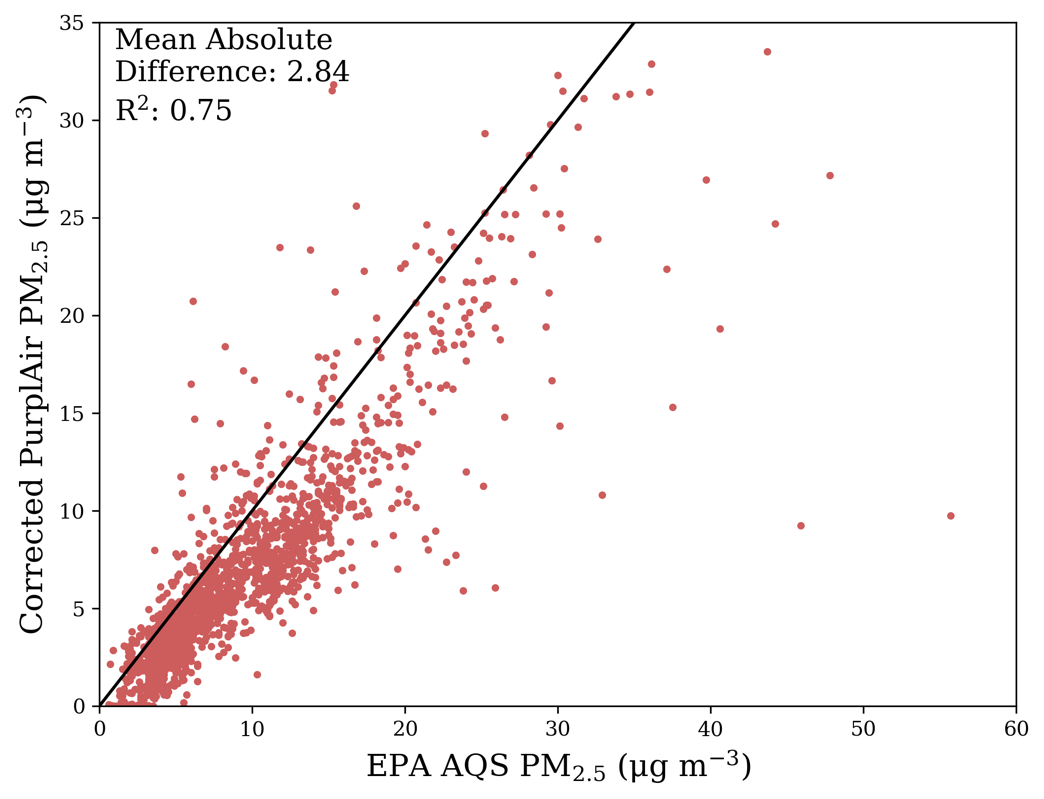


**Figure S4.** Comparison of hourly PM_2.5_ from EPA AQS 20-133-0003 and PurpleAir 1375965. The monitors were both located in Chanute, KS and were ~1.2 km apart. With a correlation of 0.75, the corrected PM_2.5_ is quite similar to the PM_2.5_ from the regulatory monitor.

Text S5.

Smoke designation decision tree

The first factor in the decision-making process was the presence of HMS smoke plumes (Figure S5). Although the HMS product may not capture all smoke (i.e., on overcast days or at night), we found it to be the best indicator of the presence of smoke in eastern Kansas. If HMS smoke plumes were localized over the monitor (with a buffer distance), we then determined if there were HMS fire hotspots nearby and to the west of the monitor(s). if there were no fires upwind, then we designated the daily measurement as smoke-free for the day. If fires were present, then we used the monitor(s) PM_2.5_ concentration(s) (day and/or nighttime). If concentrations were elevated (> 10 µg m^-3^), we designated this as an *EK_only_* day. If the monitor PM_2.5_ concentration was not elevated (< 10 µg m^-3^), this was designated as a smoke-free day.

On days with HMS smoke plumes over the monitor(s), we determined if the overlapping smoke plume(s) originated from outside eastern Kansas or the FH counties in northern Oklahoma. If the plumes were from within eastern Kansas and the monitor PM_2.5_ concentration (day and/or nighttime) was > 10 µg m^-3^, we designated this as an *EKonly* day. If the PM_2.5_ concentration was < 10 µg m^-3^, we assigned this as a smoke-free day. If the HMS smoke plumes originated from outside of eastern Kansas or the northern Oklahoma FH counties and there were also smoke plumes originating from within eastern Kansas or northern Oklahoma, we considered this to be an *EK+T* monitoring day. If no local plumes were detected, but there were HMS fire hotspots within 75 km of the monitor(s), we designated this as a *Transported Smoke Impacted* day. When there were no fire hotspots, we considered the monitor(s) with PM_2.5_ concentrations > 10 µg m^-3^ as an *EK+T* monitoring day and the monitors(s) with PM_2.5_ concentrations < 10 µg m^-3^ to be a *Transported Smoke* day.

The HMS product does not capture every smoke plume, thus, for days with no overlapping smoke plumes, we relied on several other data sources. If PM_2.5_ concentrations were low (<10 µg m^-3^) or high concentrations were centered on an urban area (Kansas City, Wichita, or Topeka), then these were designated as *Smoke-free* days. If the concentration was elevated (>10 µg m^-3^) at an isolated location and there were nearby hotpots, this was designated as *EK_only_.*

If PM_2.5_ concentrations were elevated across the region, we used coarse PM concentrations from the 9 EPA AQS monitors in the region and observations from the Kansas Automated Surface Observing System in Emporia, KS (EMP) to determine if it was a dust-impacted day. When coarse PM concentrations were >25% than the 2022 seasonal average for each individual monitor, the measurement was designated as impacted by dust and *Smoke-free.*

If PM_2.5_ concentrations were elevated across the region, but not coarse PM concentrations, we designated as *Transported Smoke* *Impacted* if there was clear transported smoke in the previous day of monitoring. If there were also fire hotspots in the region, we designated these measurements as *EK+T*. For these days without HMS plumes, we additionally used AOD and True-Color imagery from GOES to better inform our decision-making. We used daily averages of AOD (Zhang et al., 2020) and multi-step animation of AOD images from NOAA NESDIS AerosolWatch (https://www.star.nesdis.noaa.gov/smcd/spb/aq/AerosolWatch/). We also viewed animations of GeoColor from AerosolWatch.

**
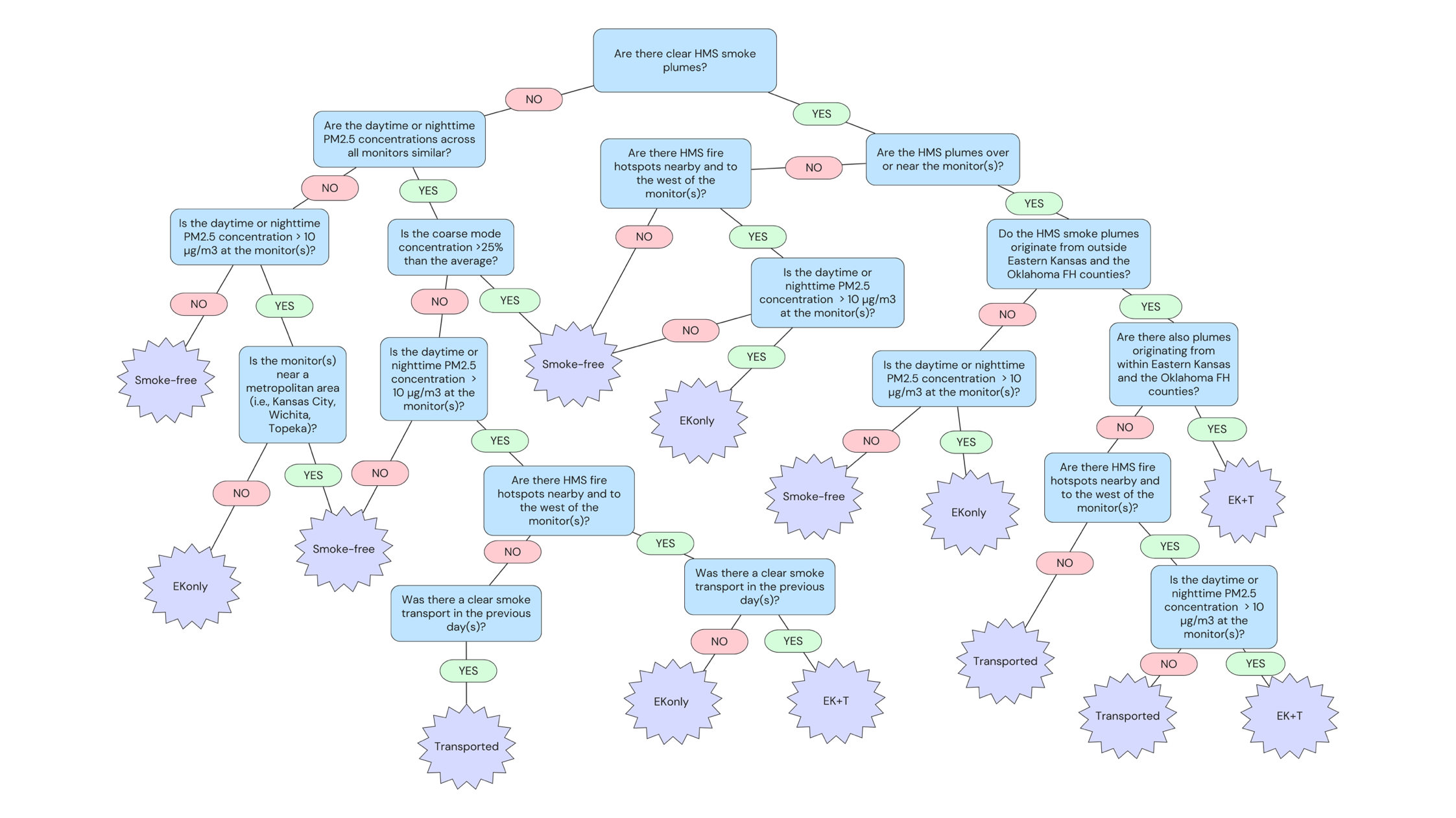
**

**Figure S5.** Decision tree for categorization of smoke.

Text S6.

Grouping of monitors for smoke designation

We grouped nearby monitors and categorized the smoke impact of them together (Figure S6). Monitors in close proximity had a similar smoke impact and could therefore be categorized as one group. Grouped monitors had an average correlation of 0.72.

**
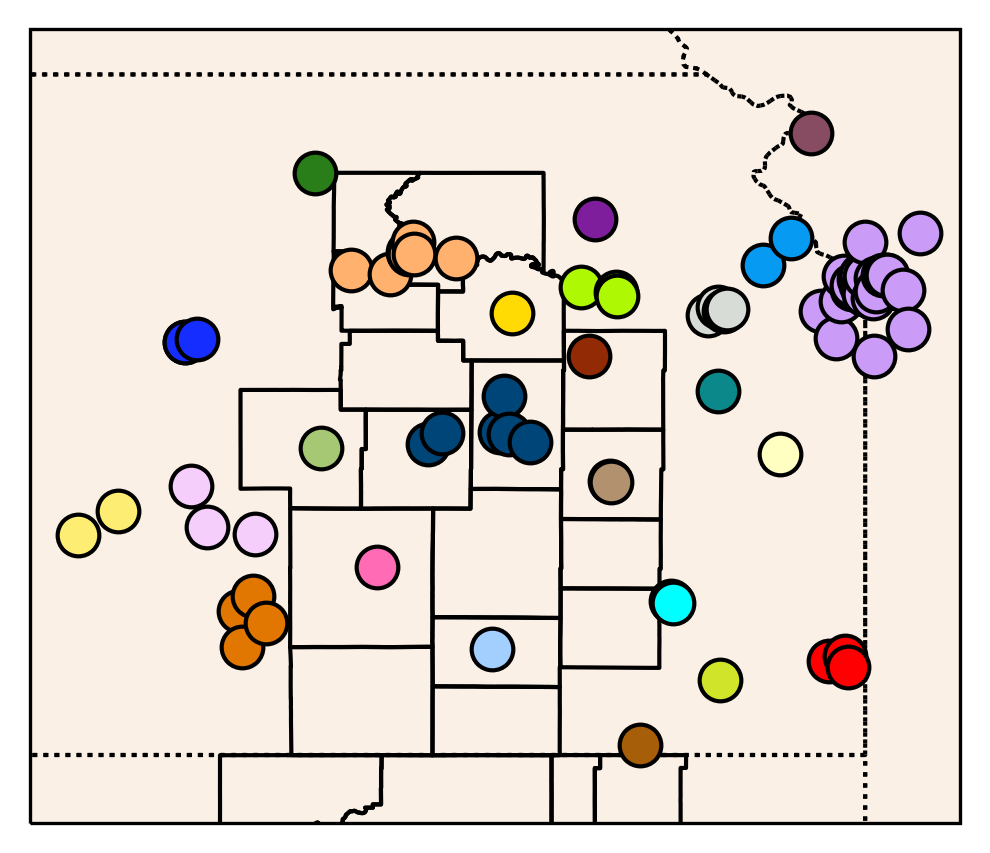
**

**Figure S6.** Grouping of monitors for smoke designation.

Text S7.

To evaluate our grouping of monitors, we calculated the correlation and mean absolute difference between the PM_2.5_ concentration from each monitor in a group (Figure S7). The grouped monitors had a higher median correlation and a lower median absolute difference during the campaign. There were three monitors with a correlation <0.4. These three monitors were located in the Kansas City area, and we expect there to be more variability in PM_2.5_ concentrations in urban areas.


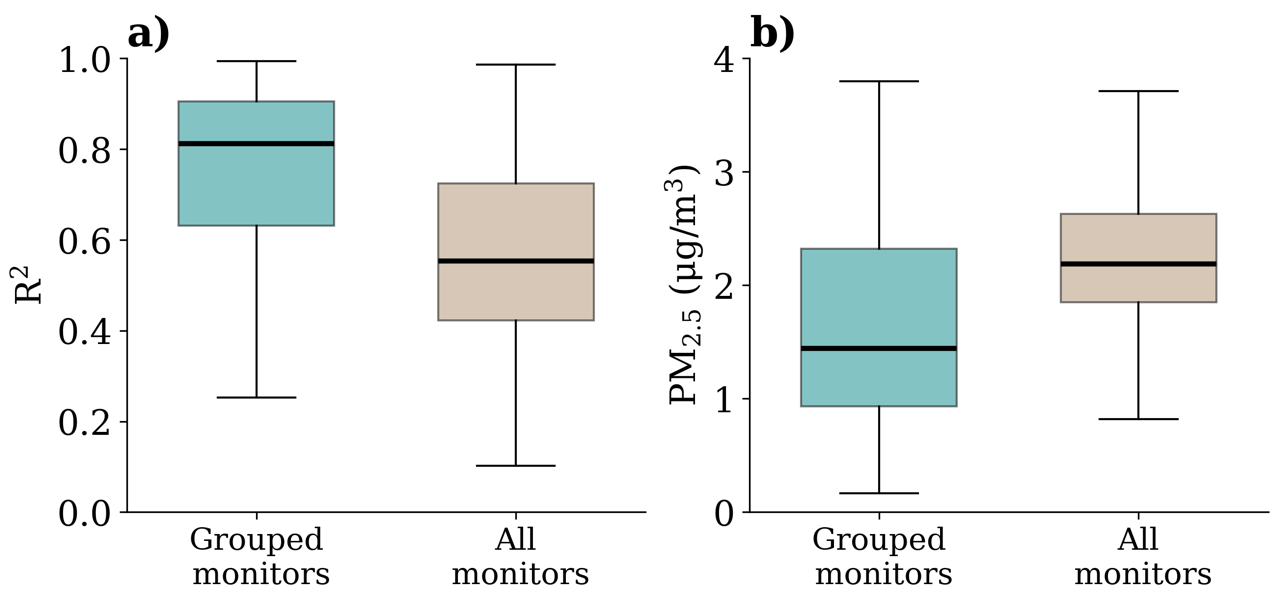


Figure S7. Correlation a) and mean absolute difference b) for the grouped monitors (blue) in comparison to the statistics for all of the monitors (beige).

Text S8.

Smoke transported from New Mexico

During our field campaign, New Mexico experienced two of the state’s largest wildfires in history (Calf Canyon/Hermits Peak). The wildfires and our campaign overlapped from 6 April to 31 May 2022. This event transported a considerable amount of smoke to eastern Kansas. For example, on 23 April 2022, there were no local fire hotspots identified by HMS in eastern Kansas; however, there were smoke plumes over the area (Figure S8). There are many fire hotspots in northern New Mexico, which likely was the source of this smoke.


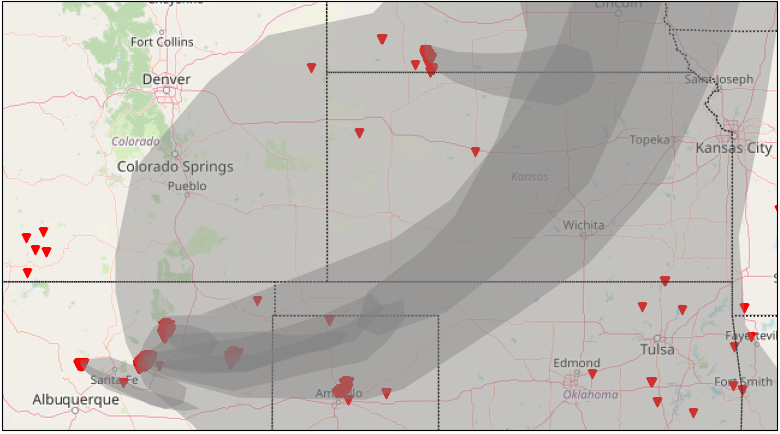


Figure S8. HMS smoke plumes and fire hotspots for 23 April 2022 during the Calf Canyon/Hermits Peak wildfire in New Mexico.

Text S9.

The Calf Canyon/Hermits Peak wildfires persisted for months and overlapped with our campaign for 55 days. Figure S9 shows the percentage of these days where there were HMS smoke plumes over the mapped area. New Mexico (including eastern Kansas) experienced a high percentage of days with smoke overhead.
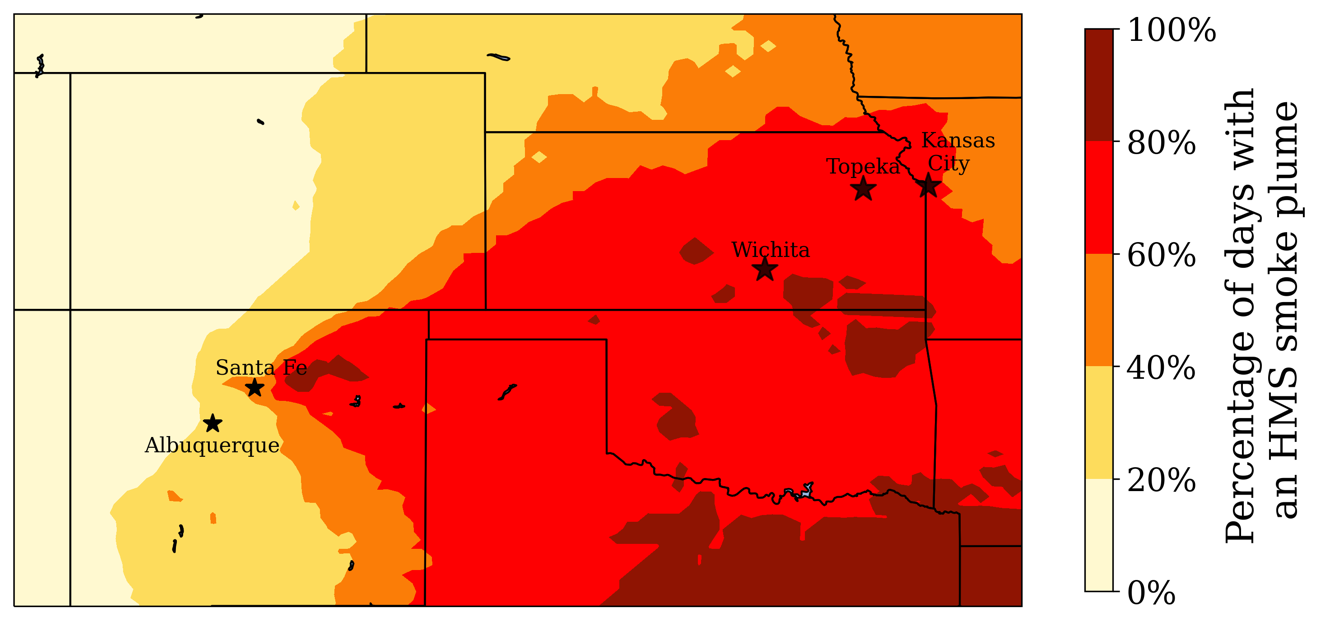


Figure S9. Percentage of days with an HMS smoke plume during the Hermit’s Peak/Calf Canyon New Mexico wildfire and our campaign (6 April to 31 May 2022).

Text S10.

Satellite product comparison to in situ measurements

We compared seasonal average PM_2.5_ concentrations from in situ measurements to two satellite products (van Donkelaar et al., 2021; Zhang & Kondragunta, 2021). The mean percent difference across all sites for the V5GL04 Monthly Hybrid product was 3% and for the NOAA GOES GWR product was 29% for the seasonal average 2022 PM_2.5_ in the FH. The correlation was low between the satellite products and the surface

measurements, with R^2^ = 0.02 (V5GL04 Monthly Hybrid) and R^2^ = 0.03 (NOAA GOES GWR).


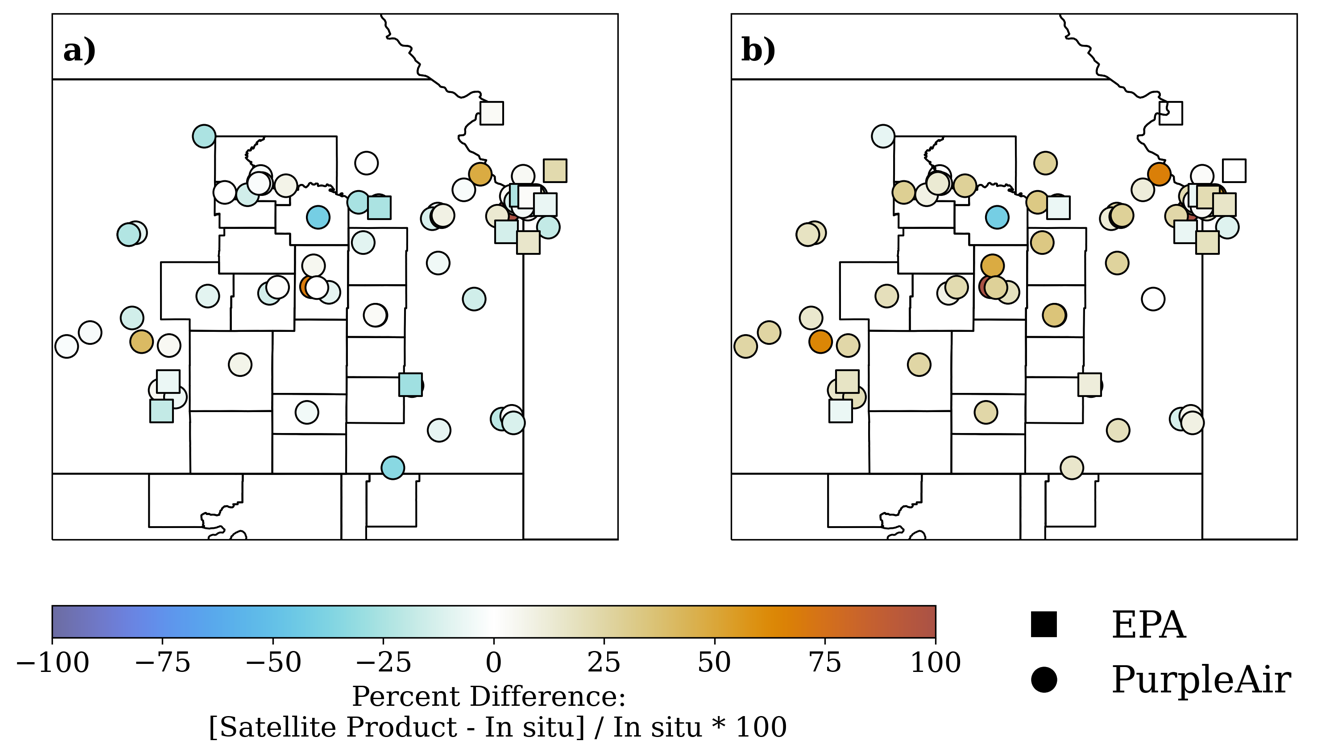


Figure S10. Percent difference between the 2022 average seasonal in situ PM_2.5_ concentrations and PM_2.5_ estimates from the a) V5GL04 Monthly Hybrid product and the b) NOAA GOES GWR product.

Text S11.

We compared the two satellite products for the past years of the FH burning season (March - May). The NOAA GOES GWR product estimates higher PM_2.5_ in the FH region than the V5GL043 Monthly Hybrid PM_2.5_ product across all three burning seasons.


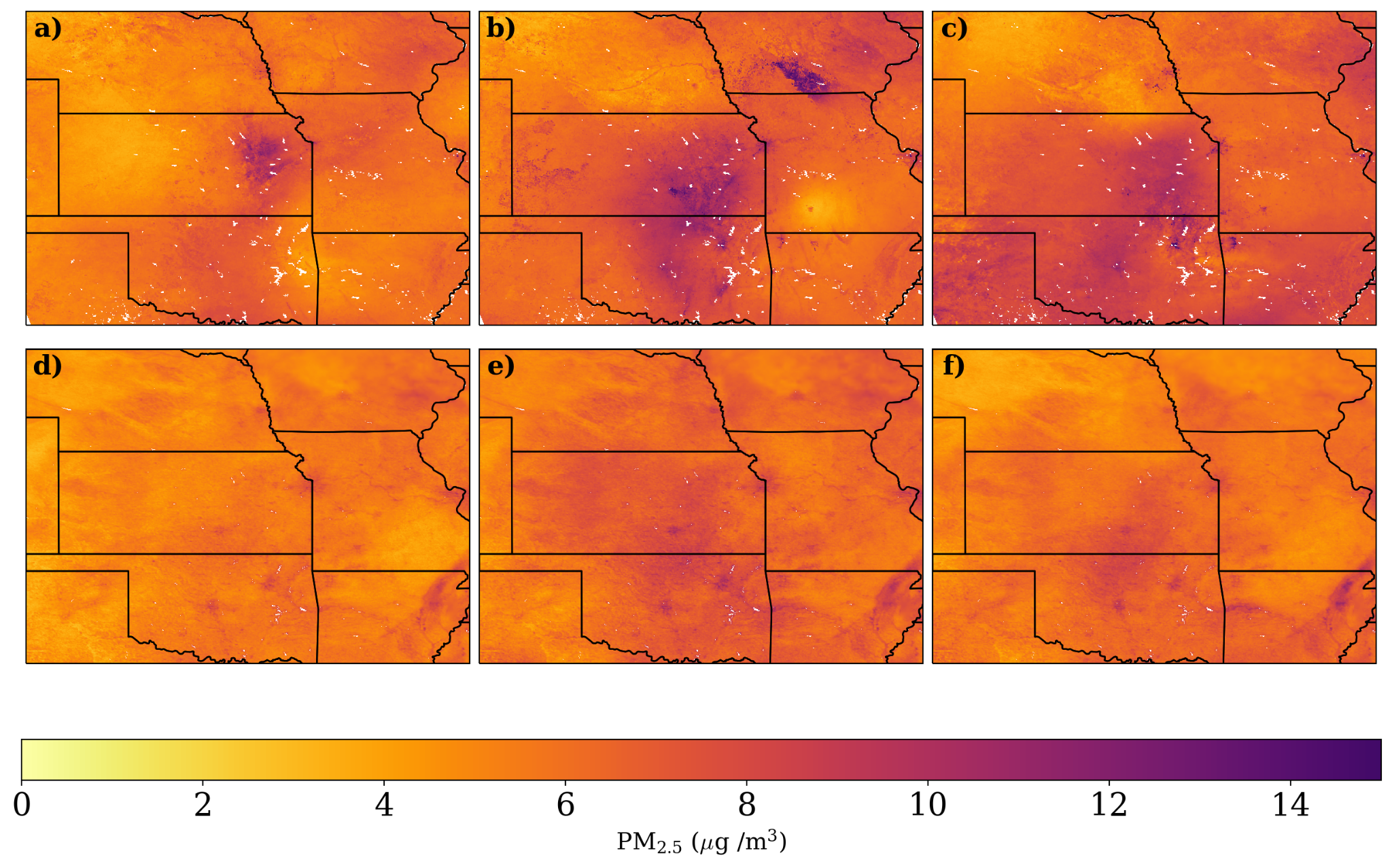


Figure S11. Comparison of seasonal averages (March - May) of the NOAA GOES PM_2.5_ product for 2020 (a), 2021 (b), and 2022 (c) and the V5GL04 Monthly Hybrid PM_2.5_ product for 2020 (d),2021 (e), and 2022 (f).
